# Peptide-functionalized periodic mesoporous silica nanoparticles for monocyte-specific TET3 Silencing enhance cardiac repair after acute myocardial infarction

**DOI:** 10.1186/s12951-025-03782-4

**Published:** 2025-11-26

**Authors:** Hao Jin, Jiandong Ding, Xiaoguo Zhang, Shouquan Cheng, Yahao Zhang, Yong Wu, Cihui Liu, Sirui Yang, Anjian Zhang, Genshan Ma, Wenbin Lu

**Affiliations:** 1 Department of Cardiology, Zhongda Hospital Affiliated with Southeast University, Nanjing, 210009 China; 2 Department of Cardiology, The Affiliated Hospital of Xuzhou Medical University, Xvzhou, 221006 China; 3 Department of Biomedical Sciences, Nanjing Normal University, Nanjing, 210023 China

## Abstract

Modulating the balance between pro- and anti-inflammatory monocyte subsets holds therapeutic promise in acute myocardial infarction (AMI); however, effective and selective strategies are still lacking. In this study, we are the first to identify Ten-Eleven-Translocation 3 (TET3) expression in circulating monocytes as an independent predictor of AMI occurrence and patient prognosis in a clinical cohort. Building on this novel insight, we engineered a monocyte-targeted RNAi delivery system designed to silence TET3 expression selectively. The platform employs periodic mesoporous silica nanoparticles (PMS) loaded with siTET3, and is surface-modified with polyethylenimine (PEI) and polyethylene glycol (PEG) to enhance cellular uptake. Critically, we further functionalized the system with a CD14 receptor-recognizing transmembrane peptide (Cys-Gly-Trp-Arg-Arg-Arg-NH₂), enabling precise monocyte targeting and internalization. Our targeted nanotherapeutic successfully reprogrammed inflammatory monocytes in vitro, leading to attenuated pro-inflammatory phenotypes. In vivo, treatment with siTET3-loaded nanoparticles markedly reduced infarct size and myocardial fibrosis in murine AMI models. Importantly, translational validation in a porcine AMI model demonstrated substantial suppression of cardiac inflammation and improved post-infarction outcomes following systemic administration of the nanotherapeutic.

**Graphical abstract:**

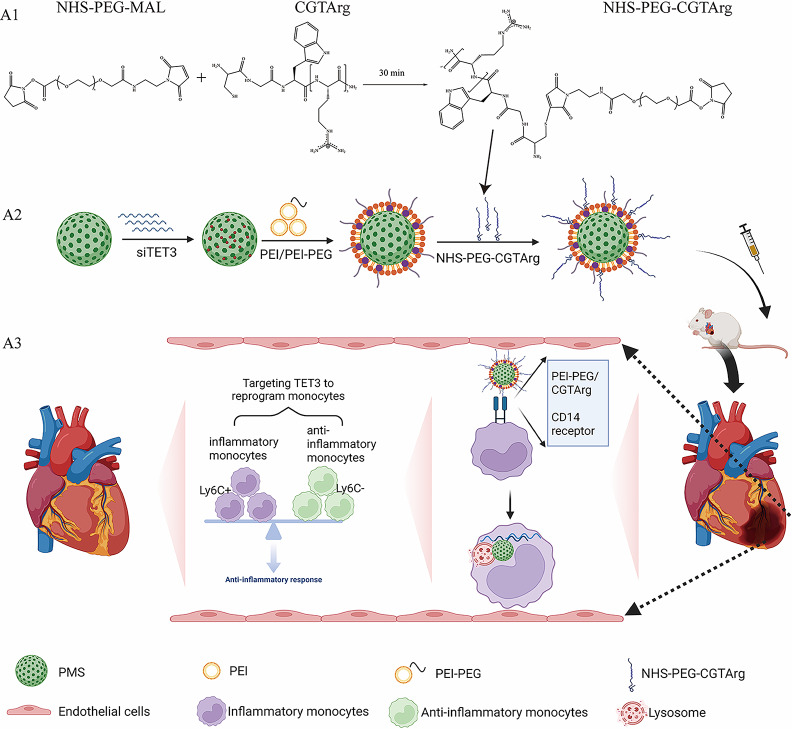

**Supplementary Information:**

The online version contains supplementary material available at 10.1186/s12951-025-03782-4.

## Background

Acute myocardial infarction (AMI), recognized as a clinical emergency and a severe condition, involved the destruction and necrosis of myocardial tissue due to continuous coronary artery obstruction. Currently, the number of patients suffering from cardiovascular diseases in China is approximately 330 million. The recent release of the “China Health and Nutrition Survey Yearbook” revealed that from 2002 to 2020, the mortality rate from AMI had generally been increasing, peaking in 2020. The China PEACE study, which surveyed 162 hospitals across 31 provinces, autonomous regions, and municipalities from 2001 to 2011, found that the hospitalization rate for STEMI, estimated per 100,000 natural population, rose from 3.7 in 2001 to 8.1 in 2006, and 15.8 in 2011 [1]. In the management of myocardial infarction (MI), antiplatelet therapy has been the predominant approach since 1980, while different interventional treatments had gained increasing prominence from 2000 [2, 3]. However, data from the German Myocardial Infarction Registry showcased a persistently high incidence and mortality of AMI, highlighting the critical need for novel treatment options [4]. The COLCOT study, which enrolled 4,745 MI patients administered with colchicine and placebo over a follow-up period of 22.6 months, revealed that colchicine exhibited a significant reduction in the risk of major myocardial infarction events (hazard ratio [HR], 0.77; 95% confidence interval [CI], 0.61–0.96; *P* = 0.02), including cardiovascular death, cardiac arrest, MI, stroke, and emergency percutaneous transluminal coronary intervention (PCI) [5], demonstrating that anti-inflammatory treatment might enhance the prognosis of MI. Nowadays, monocytes in AMI were considered to play key roles in two distinct phases, namely the pro-inflammatory phase and the anti-inflammatory phase. In the initial three days following AMI, pro-inflammatory monocytes were implicated in myocardial necrosis and cardiac remodeling in the pro-inflammatory phase. Conversely, during the anti-inflammatory stage, anti-inflammatory monocytes played a crucial role in the removal of necrotic debris, enhancement of circulation and maintenance of vascular stability [6, 7]. In humans, the corresponding markers were CD14^+^CD16^−^ for inflammatory monocytes and CD14^−^CD16^+^ for anti-inflammatory monocytes. In mice and pigs, the surface markers distinguishing inflammatory and anti-inflammatory monocytes were Ly6C and CD163, respectively [8]. Subsequent sequencing studies revealed a higher expression of Ten-Eleven-Translocation-3 (TET3) in inflammatory monocytes, while the expression was lower in anti-inflammatory monocytes. Moreover, TET3^−/−^ mice exhibited the reprogramming of the two monocytes compared to wild-type mice [9]. As a result, the therapeutic approach of silencing TET3 expression held potential in improving the prognosis of AMI by modulating reprogramming monocytes. Gene therapy, especially for siRNA therapy, emerged as a promising therapeutic platform. By now, several siRNA drugs had received approval from the US Food and Drug Administration (FDA), including alirocumab and evolocumab [10, 11]. In contrast to monoclonal antibodies, siRNA therapy exhibited superior targeting capabilities and was not influenced by the spatial structure of the protein. Nevertheless, siRNA therapy had several significant weaknesses, such as limited stability, susceptibility to hydrolysis, and unfavorable pharmacokinetics. Moreover, once inside the cell, siRNA could be degraded by lysosomes [12]. Initially developed by scientists around the year 2001, periodic mesoporous silica nanoparticles (PMS) gained popularity in the field of biomedical applications due to their unique characteristics, including customizable mesoporous structure, extensive surface area, large pore volume, selective surface functionality and controllable morphology, which have been successfully utilized for drug delivery in the medical field [13, 14]. Currently, membrane fusion and the proton sponge effect were the two primary mechanisms involved in the successful delivery of siRNA. In our study, the proton sponge effect was applied using PEI to deliver the siRNA [15, 16]. Corresponding research also indicated that relying solely on the membrane fusion mechanism, although achieving targeted contact with monocytes, makes it challenging to successfully deliver siRNA into monocytes [17]. Therefore, in this study, we relied on the proton sponge effect formed by PEI to achieve targeted delivery of siRNA. The existing research has revealed that monocytes relied on CD14 for the phagocytosis of foreign particles and bacteria. Moreover, peptide modifications with arginine (Arg) and tryptophan (Trp) were shown to enhance the targeted delivery of nanoparticles into monocytes. Building upon this, Münteret al. engineered lipidosome with surface modifications using polyethylene glycol (PEG), Arg and Trp, successfully targeting monocytes without interference with other cells in the bloodstream [18].

**Fig. 1:**
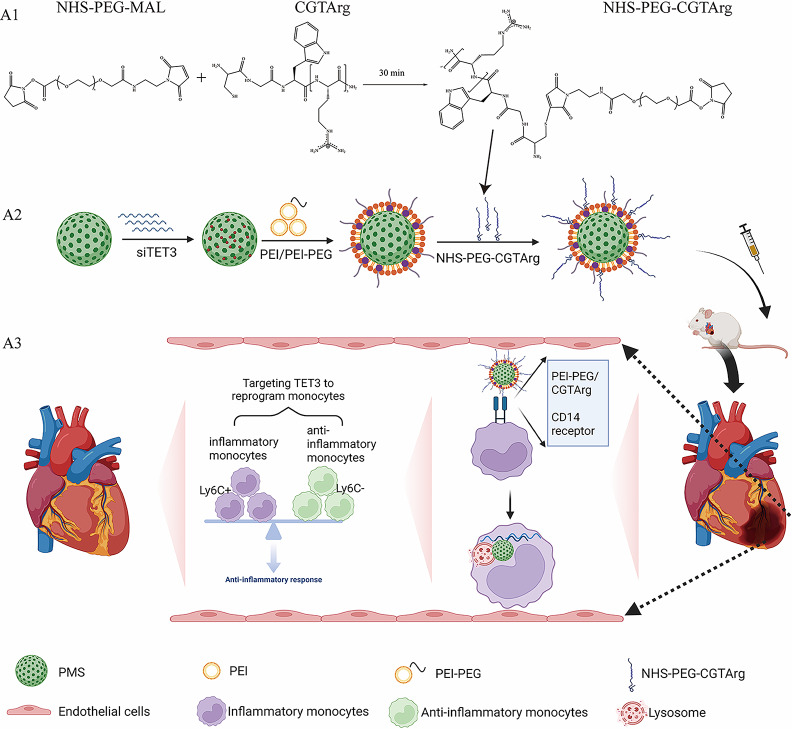
Schematic illustration of the preparation of PMS-siTET3-PEI-PEG/PEI-PEG-CGTArg for reprogramming monocytes. **A1**-**A2**: Schematic diagram of the fabrication process of PMS-siTET3-PEI-PEG/PEI-PEG-CGTArg; **A3**: PMS-siTET3-PEI-PEG/PEI-PEG-CGTArg contributing to the reprogramming of monocytes and improving the prognosis of myocardial infarction in AMI mice with the help of Biorender (https://www.biorender.com/)

In light of this, we developed the delivery system targeting monocytes with PMS and siTET3 (siRNA targeting TET3) as the main components, along with surface modifications using the peptides (Cys-Gly-Trp-Arg-Arg-Arg-NH_2_) and PEG. The particles aimed to reprogram the monocytes based on targeting CD14 receptor, thereby enhancing the prognosis of AMI (Fig. 1A1-A3).

## Result

### Research on human

1. **TET3 expression in AMI based on the GEO database**

**Fig. 2:**
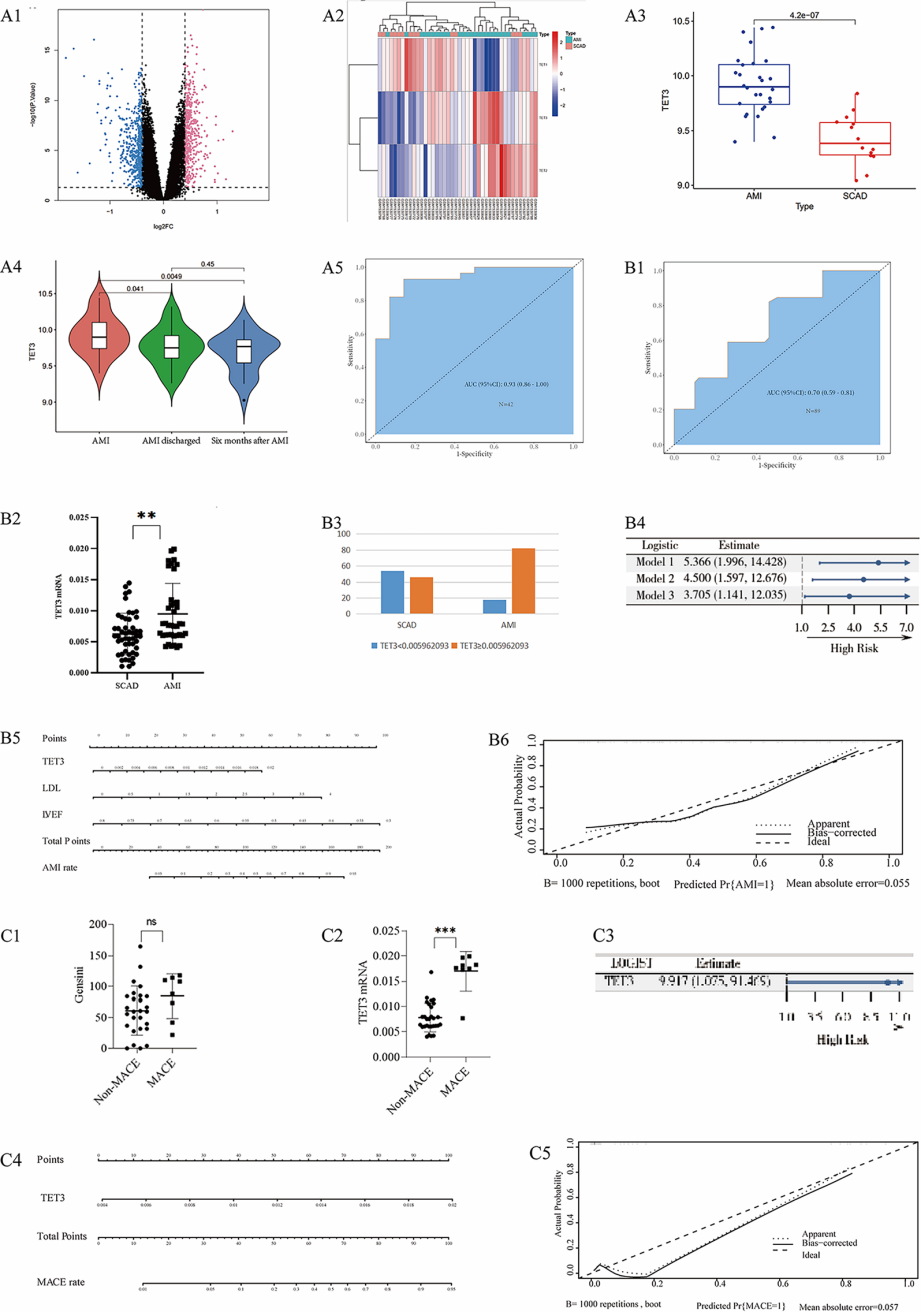
TET3 expression differences in human. **A1**: Volcano plot of gene differences between SCAD and AMI patients (Red indicating upregulated genes, blue indicating downregulated genes, and black indicating non-differential genes); **A2**: Heatmap of TET family gene differences between SCAD and AMI patients, including TET1, TET2, and TET3; **A3**: Differential expression analysis of TET3 between AMI and SCAD patients; **A4**: Variations in TET3 expression in AMI patients across different phases: at admission, discharge, and 6 months post-AMI; **A5**: TET3 served as a diagnostic marker for AMI risk based on GEO data; **B1**: Diagnosis of AMI occurrence based on monocyte TET3 expression levels; **B2**: Comparison of monocyte TET3 expression differences between the SCAD and AMI groups (TET3 as a continuous variable); **B3**: Comparison of monocyte TET3 expression differences between the SCAD and AMI groups (TET3 as a categorical variable); **B4**: Logistic regression analysis for the risk of AMI; **B5**: Nomogram prediction model for AMI risk;B6: Calibration curve for the nomogram predicting AMI risk; **C1**: Comparison of Gensini scores between the Non-MACE and MACE groups; **C2**: Comparison of monocyte TET3 expression differences between the Non-MACE and MACE groups (TET3 as a continuous variable); **C3**: Logistic regression analysis of MACE risk in AMI patients; **C4**: Nomogram prediction model for MACE risk in AMI patients; **C5**: Calibration curve for the nomogram predicting MACE risk in AMI patients. (N=3, ns p>0.05, *p<0.05, **p<0.01,***p<0.001)

Based on the acquired GEO dataset, our study utilized the R programming language with the ggplot2 package to identify all highly expressed and lowly expressed genes, generating a volcano plot. A total of 963 differentially expressed genes were discovered, comprising 433 upregulated genes and 530 downregulated genes. Notably, TET3, the gene of interest in our study, was found to be upregulated (Fig. 2A1). Considering the focus on the TET family, differential gene expression within the TET family, including TET1, TET2, and TET3, was presented using a heatmap analysis. The results revealed that within the TET family, particularly for the TET3 gene, it was observed that TET3 exhibited higher expression in AMI patients and lower expression in SCAD patients (Fig. 2A2). Further investigation using the R ggpubr package employed boxplots to illustrate the expression differences of TET3 between AMI and SCAD patients. The findings indicated that TET3 was highly expressed in AMI patients (Fig. 2A3). Relative to post-AMI levels and 6-month follow-up, TET3 expression was upregulated during acute AMI (Fig. 2A4). In addition, TET3 also exhibited superior diagnostic performance for AMI detection (Fig. 2A5) (AUC = 0.93, 95CI%: 0.86–1.00).

2) **Association between TET3 expression and AMI risk based on clinical data**

The baseline data results are shown in Table 1. A comparison between the two groups revealed no statistically significant differences (*P* > 0.05) in terms of general clinical characteristics (including gender, age, family history, and smoking history), comorbidities (hypertension, HLP, DM, and CKD), blood test parameters (Hb, BUN, Cr, UA, TG, TCHO, HDL-C), or medication use (statins, antiplatelet agents, ACEI/ARB, β-blockers, and calcium channel blockers). As shown in Fig. 2B1 (AUC = 0.70, 95CI%: 0.59–0.81), TET3 exhibited superior diagnostic performance for AMI detection. As shown in Fig. 2B2, monocytes from AMI patients exhibited significantly higher TET3 mRNA expression levels compared to those from the SCAD group (0.009493 ± 0.0049098 vs. 0.006251 ± 0.0033465, *P* = 0.001). Furthermore, based on the optimal cutoff value of 0.005962093 for TET3 in diagnosing AMI risk, this study converted TET3 expression from a continuous variable to a categorical variable, defining TET3 ≥ 0.005962093 as high expression and TET3 < 0.005962093 as low expression. As shown in Fig. 2B3, chi-square analysis revealed that the AMI group had a significantly higher proportion of patients with high TET3 expression (82.1% vs. 46%), with the difference being statistically significant (*P* = 0.001). Compared to low TET3 levels, high TET3 expression was associated with an increased risk of AMI (OR: 5.366, 95% CI: 1.996–14.428, *P* = 0.001). Furthermore, a multivariate regression analysis was performed to adjust for hematological parameters with significant differences. The results showed that high TET3 levels remained an independent risk factor for AMI (OR: 4.500, 95% CI: 1.597–12.676, *P* = 0.004). Subsequently, a Logistic regression analysis was conducted to adjust for all significant variables. The findings confirmed that high TET3 levels continued to be an independent predictor of AMI risk (OR: 3.705, 95% CI: 1.141–12.035, *P* = 0.029) (Fig. 2B4). Based on the results of the Logistic regression analysis, a nomogram was constructed to predict AMI risk. As shown in Fig. 2B5, TET3 played a crucial role in predicting AMI occurrence. Furthermore, calibration curve analysis and the Hosmer-Lemeshow goodness-of-fit test demonstrated that the predicted incidence of AMI closely matched the actual incidence, indicating a well-fitted model (Fig. 2B6).

**Table 1:** Baseline characteristics in patients with AMI or SCAD

	SCAD (*n* = 50)	AMI (*n* = 39)	*P*
Age, y	64.9 ± 9.20	62.6 ± 15.8	0.417
Hb, g/l	136 (128, 149)	132 (120, 150)	0.445
LVEF, %	0.69 (0.62, 0.71)	0.56 (0.43, 0.67)	**< 0.001**
Bun, mmol/l	6.30 (5.60, 7.10)	5.30 (4.55, 7.30)	0.051
Cr, mmol/l	69.0 (58.0, 81.0)	77.0 (60.0, 93.5)	0.156
UA, mmol/l	301 (262, 362)	300 (233, 417)	0.960
TG, mmol/l	1.20 (0.92, 1.90)	1.48 (1.02, 2.12)	0.135
TCHO, mmol/l	3.80 ± 1.04	4.16 ± 1.10	0.122
LDL-C, mmol/I	1.99 ± 0.798	2.38 ± 0.689	**0.02**
HDL, mmol/l	0.99 (0.84, 1.15)	0.89 (0.72, 1.08)	0.058
Male, %	60.0	69.2	0.368
Family history, %	4.1	2.7	0.605
Smoking, %	34.0	51.3	0.101
Hypertension, %	80.0	66.7	0.154
DM, %	42.0	39.5	0.811
HLP, %	2.0	7.7	0.221
CKD, %	4.0	15.4	0.068
Statins, %	100	100	1.00
Antiplatelet drugs, %	94.0	100	0.185
ACEI, %	56.0	73.0	0.105
Β receptor blockers, %	74.0	73.0	0.914
Ca^2+^channel blockers, %	16.0	8.1	0.223

Bold values indicate a statistically significant difference (*P* < 0.05)

Hemoglobin: Hb; blood urea nitrogen: BUN; creatinine: Cr; uric acid: UA; triglycerides: TG; total cholesterol: TCHO; high-density lipoprotein cholesterol: HDL-C; low-density lipoprotein cholesterol: LDL-C; left ventricular ejection fraction: LVEF; diabetes mellitus: DM; hyperlipidemia: HLP; chronic kidney disease: CKD; angiotensin-converting enzyme inhibitors: ACEI

3) **Association between TET3 expression and AMI prognosis based on clinical data**

In this study, a total of 39 AMI patients were enrolled. Two patients were lost to follow-up, as they were discharged voluntarily and did not complete in-hospital follow-up. Subsequently, the remaining 37 patients successfully completed in-hospital follow-up. The follow-up results showed that 8 patients experienced MACE events, including 1 case of mortality and 7 cases of cardiogenic shock or acute left heart failure.

There were no statistically significant differences (*P* > 0.05) between the Non-MACE and MACE groups in terms of general clinical characteristics (including sex, age, smoking history, and family history), comorbidities (including hypertension, HLP, DM, and CKD), blood test results (including Hb, BUN, Cr, UA, TG, TCHO, HDL-C, and LDL-C), echocardiographic findings (including LVEF), and medication use (including statins, antiplatelet agents, ACEI/ARB, β-blockers, and Ca²⁺ channel blockers) (Table 2). As shown in Fig. 2C1, there was no significant difference in Gensini scores between the two groups (84.5 ± 35.9 vs. 61.2 ± 39.5, *p* = 0.145) (Fig. 2C1). Compared to patients in the Non-MACE group, those in the MACE group exhibited significantly higher TET3 mRNA expression levels in monocytes (0.01697 ± 0.0038920 vs. 0.007775 ± 0.0077749, *p* < 0.001) (Fig. 2C2). Furthermore, Logistic regression analysis was performed on the identified differential factors, revealing that higher TET3 mRNA expression was associated with an increased risk of MACE in AMI patients compared to lower TET3 mRNA expression (OR: 9.917, 95% CI: 1.075–91.469, *p* = 0.043) (Fig. 2C3). Based on the results of Logistic regression analysis, this study constructed a nomogram to predict the MACE risk in AMI patients. As shown in Fig. 2C4, the TET3 expression level in monocytes played a significant role in predicting the risk of MACE. Further, calibration curve analysis and the Hosmer-Lemeshow goodness-of-fit test revealed that the predicted MACE incidence from the model closely aligned with the actual MACE incidence in patients, demonstrating good model fit (Fig. 2C5).

**Table 2:** Baseline characteristics in patients

	Non-MACE (*n* = 29)	MACE (*n* = 8)	*P*
Age, y	60.4 ± 15.0	65.6 ± 16.7	0.407
Hb, g/l	136 ± 21.5	131 ± 20.6	0.59
LVEF	0.559 ± 0.140	0.498 ± 0.115	0.330
Bun, mmol/l	5.20 (4.40, 6.60)	8.60 (5.12, 14.9)	0.080
Cr, mmol/l	75.0 (58.0, 89.0)	93.5 (67.0, 219)	0.216
UA, umol/I	310 ± 124	380 ± 170	0.216
TG, mmol/l	1.35 (1.01, 2.12)	1.73 (1.42, 1.78)	0.472
TCHO, mmol/l	4.15 ± 1.15	3.98 ± 0.974	0.715
LDL-C, mmol/I	2.35 ± 0.704	2.40 ± 0.716	0.855
HDL, mmol/l	0.91 (0.74, 1.08)	0.88 (0.71, 0.89)	0.230
Male, %	72.4	75.0	0.633
Family history, %	3.4	0	0.829
Smoking, %	55.2	50.0	0.553
Hypertension, %	62.1	75.0	0.408
DM, %	31.0	71.4	0.064
HLP, %	10.3	0	0.47
CKD, %	13.8	25.0	0.387
Statins, %	100	100	1.00
Antiplatelet drugs, %	100	100	1.00
ACEI, %	74.1	75.0	0.670
Β receptor blockers, %	74.1	87.5	0.396
Ca^2+^channel blockers, %	7.4	12.5	0.553

### Synthesis and characterization of PMS-siTET3-PEI-PEG/PEI-PEG-CGTArg

#### Loading of siTET3 in the particles and evaluation of the system stability

**Fig. 3:**
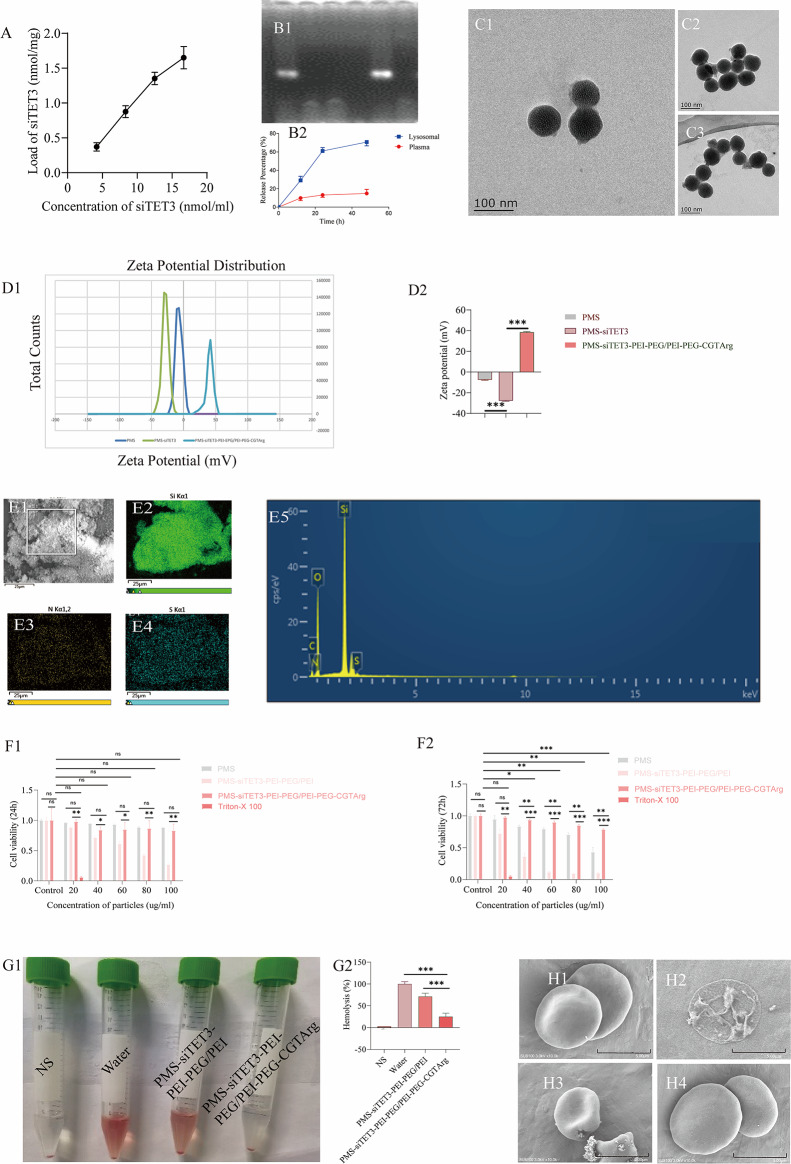
Characterization and biocompatibility of the particles. **A**: Adsorption capacity of PMS with increasing concentrations of siTET3 in solution; **B1**: Stability of the delivery system assessed via electrophoretic analysis of agarose gels; TEM images of PMS with different modifications; **B2**: Release of siTET3 from the nanoparticles in simulated plasma and lysosomal environments; **C1**: PMS; **C2**: PMS-siTET3-PEI-PEG/PEI; **C3**: PMS-siTET3-PEI-PEG/PEI-PEG-CGTArg; **D1**: Zeta potential of the PMS with different modifications; **D2**: Comparison of Zeta potential of the PMS with different modifications; **E1**: SEM image of PMS-siTET3-PEI-PEG/PEI-PEG-CGTArg; **E2**-**E5**: EDS characterization of PMS-siTET3-PEI-PEG/PEI-PEG-CGTArg; The cell viability of THP-1 cells incubated with varying concentrations of different particles and Triton X-100 for 24h (**F1**) and 72h (**F2**); **G1**: Hemolysis images of different particles (including NS, Water, PMS-siTET3-PEI-PEG/PEI, PMS-siTET3-PEI-PEG/PEI-PEG-CGTArg) incubated with RBCs; **G2**: Statistical results of hemolysis with different particles incubated with RBCs; SEM images of RBCs treated with NS (**H1**), water (**H2**), PMS-siTET3-PEI-PEG/PEI (**H3**) and PMS-siTET3-PEI-PEG/PEI-PEG-CGTArg (**H4**). (N=3, ns p>0.05, *p<0.05, **p<0.01, ***p<0.001)

As shown in Fig. 3 A, the amount of siTET3 loaded in the delivery systems increased with the concentrations of siTET3 increasing.

An agarose gel electrophoresis analysis was conducted to assess the protective effect of the delivery system on siTET3 degradation. In Fig. 3B, Group 1: siTET3 was dispersed in water. During electrophoresis, bands were observed on the gel, indicating that siTET3 could be detected via electrophoresis; Group 2: siTET3 was dispersed in water and incubated with RNase for 30 min. No bands were observed, indicating that siTET3 was degraded by RNase; Group 3: PMS-siTET3-PEI-PEG/PEI-PEG-CGTArg was dispersed in water. No bands were observed, indicating that siTET3 was protected inside PMS-siTET3-PEI-PEG/PEI-PEG-CGTArg; Group 4: PMS-siTET3-PEI-PEG/PEI-PEG-CGTArg was dispersed in water and incubated with RNase for 30 min. No bands were observed, indicating that PMS-siTET3-PEI-PEG/PEI-PEG-CGTArg was co-incubated with RNase without release of siTET3; Group 5: PMS-siTET3-PEI-PEG/PEI-PEG-CGTArg was treated with RNase and then incubated with heparin for 30 min. Bands corresponding to siTET3 were observed, indicating that after PMS-siTET3-PEI-PEG/PEI-PEG-CGTArg was treated with RNase, the siTET3 inside was protected from degradation and could be released and detected. This demonstrated that PMS-siTET3-PEI-PEG/PEI-PEG-CGTArg could protect the encapsulated siTET3 from RNase degradation; Group 6: PMS-siTET3-PEI-PEG/PEI-PEG-CGTArg was treated with RNase and then co-incubated with both heparin and RNase for 30 min. No bands were observed, indicating that the siTET3 released from PMS-siTET3-PEI-PEG/PEI-PEG-CGTArg was degraded by RNase, demonstrating that the siRNA still retained its characteristic.

In summary, these results indicated that PMS-siTET3-PEI-PEG/PEI-PEG-CGTArg could protect the encapsulated siTET3 from RNase degradation.

In addition, we investigated the siRNA release efficiency under different conditions. As shown in the Fig. 3B2, in the simulated plasma environment, the release rate of siRNA remained relatively low within 48 h. In contrast, in the simulated lysosomal environment, the release efficiency of siRNA was significantly higher, with a substantial release observed within the first 24 h, followed by a relatively slower release thereafter. These results indicate that the nanoparticle system is relatively stable and capable of releasing siRNA effectively in the lysosomal environment.

#### Characterization of PMS-siTET3-PEI-PEG/PEI-PEG-CGTArg

TEM observations (Fig. 3C1) revealed homogeneous particles and pores in the unmodified PMS. However, after modification with PEI/PEI-PEG, PMS particles were observed to be surrounded by PEI/PEI-PEG (Fig. 3C2), leading to poor dispersion. Subsequent modification with CGTArg resulted in improved dispersion (Fig. 3C3). Moreover, the surface potential of the particles became positive after modification with PEG/PEI-PEG and CGTArg (Fig. 3D1 and Fig. 3D2). In addition, 1 mg PMS-siTET3-PEI-PEG/PEI-PEG-CGTArg was modified with 26.6 µg CGTArg.

Based on SEM, EDS scanning revealed the presence of Si, N and S elements. Si was a characteristic element of PMS, while N and S were characteristic elements of the targeted peptide, thus confirming that the CGTArg peptide was successfully modified onto PMS (Fig. 3E1-E5).

### Research on cells

#### Evaluation the biocompatibility and transfection efficiency of PMS-siTET3-PEI-PEG/PEI-PEG-CGTArg

1. **Toxicity of PMS-siTET3-PEI-PEG/PEI-PEG-CGTArg to monocytes**

The results showed that Triton X-100 exhibited significant toxicity to the cells. Monocytes were incubated with different particles (including PMS, PMS- siTET3- PEI-PEG/PEI and PMS-siTET3-PEI-PEG/PEI-PEG-CGTArg) for 24 and 72 h. The viability of monocytes decreased with the concentrations of particles increasing. Notably, PMS at different concentrations did not show significant toxicity after 24h of incubation. In contrast, PMS-siTET3-PEI-PEG/PEI exhibited evident cytotoxicity towards cells at higher concentrations, especially after longer incubation times. After modified with CGTArg, PMS-siTET3-PEI-PEG/PEI-PEG-CGTArg showed minimal cytotoxicity at a lower concentration after being modified with CGTArg (Fig. 3F1 and Fig. 3F2).

2) **Red blood cell biocompatibility of PMS-siTET3-PEI-PEG/PEI-PEG-CGTArg**

Since the particles would be injected into the bloodstream, the effect of particles on RBCs was also evaluated. After particles incubation with RBCs for 2 h, hemolysis was observed with PMS- siTET3- PEI-PEG/PEI compared to NS and PMS-siTET3-PEI-PEG/PEI-PEG-CGTArg (Fig. 3G1-G2). Additionally, RBCs associated with different treatments were observed using SEM. After being treated with NS and PMS-siTET3-PEI-PEG/PEI-PEG-CGTArg, RBCs still maintain the normal biconcave disc shape (Fig. 3H1-H4).

3) **Transfection efficiency of PMS-siTET3-PEI-PEG/PEI-PEG-CGTArg**

Flow cytometry process for assessing nanoparticle transfection efficiency was shown in Figure S1B1-BB3. Higher concentrations (20 µg/mL, Figure S1B5) exhibited better transfection efficiency compared to lower concentrations (10 µg/mL, Figure S1B4), and this difference was statistically significant (Figure S1B6).

In addition, nanoparticles (20 µg/mL) exhibited minimal cytotoxicity. As a consequence, the concentration (20 µg/mL) was adopted for our study.

#### Targeting ability and lysosome escape of the delivery system

**Fig. 4:**
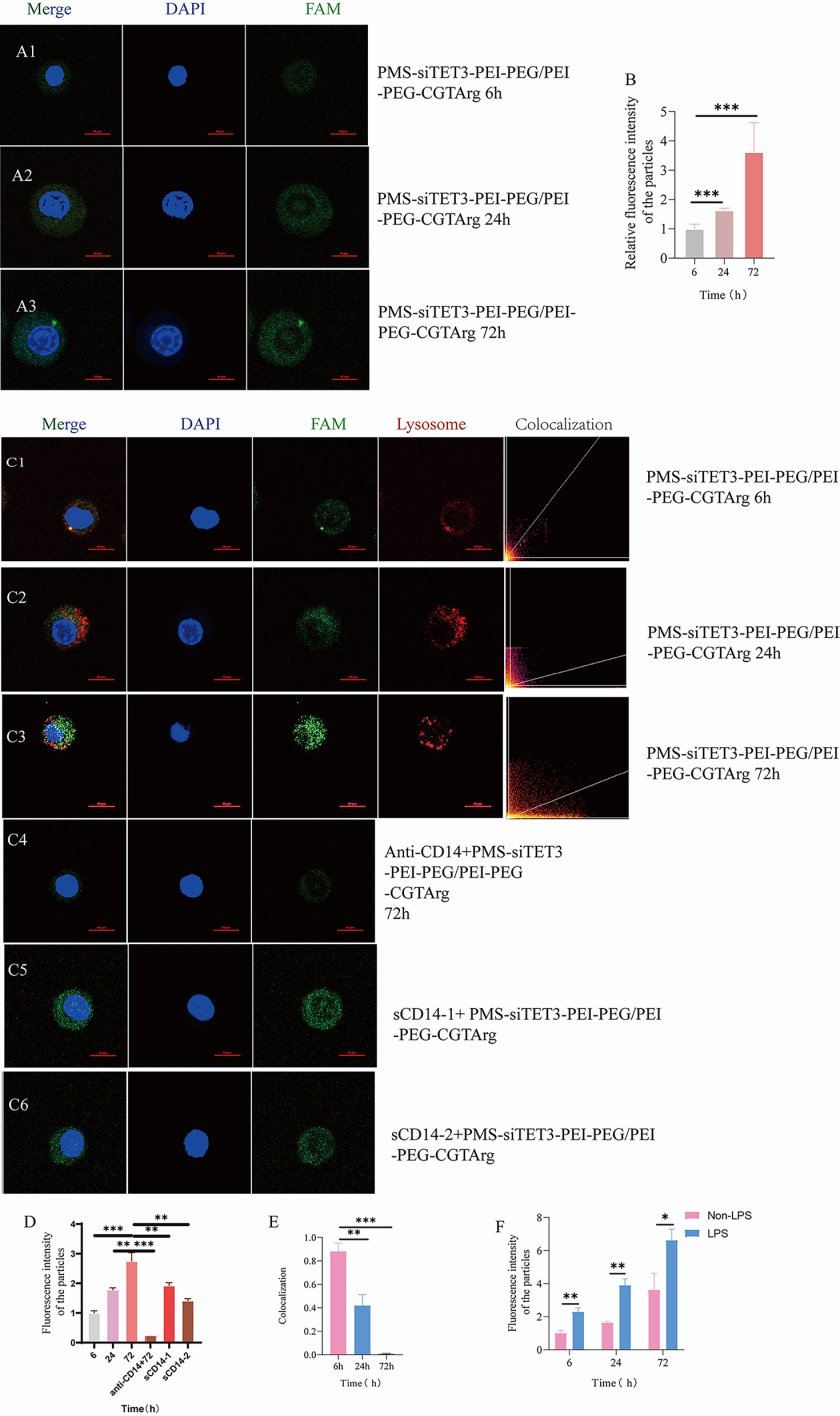
Targeting specificity and lysosomal escape of nanoparticles. Images of THP-1 cells (NOT treated with LPS) incubated with PMS-fam-siTET3-PEI-PEG/PEI-PEG-CGTArg for 6h (**A1**), 24h (**A2**), and 72h (**A3**) (blue: DAPI, green: PMS-fam-siTET3-PEI-PEG/PEI-PEG-CGTArg); **B**: Statistical comparison of the relative fluorescence intensity of nanoparticles in monocytes (NOT treated with LPS) at different time points (6h, 24h and 72h); Images of THP-1 cells (treated with LPS) incubated with PMS-fam-siTET3-PEI-PEG/PEI-PEG-CGTArg for 6h (**C1**), 24h (**C2**), 72h (**C3**) and 72h (**C4**) (THP-1 was blocked with anti-CD14 antibody before incubation with particles) (blue: DAPI, green: PMS-fam-siTET3-PEI-PEG/PEI-PEG-CGTArg, red: lysosome); Images of THP-1 cells (treated with LPS) incubated with PMS-fam-siTET3-PEI-PEG/PEI-PEG-CGTArg and sCD14 (**C5**: 500 ng/ml, **C6**: 1500 ng/ml) for 72h; **D**: Statistical comparison of the relative fluorescence intensity of nanoparticles in monocytes (treated with LPS) at different time points; **E**: Colocalization of lysosomes and particles in THP-1 cells treated with particles for 6h, 24h and 72h; **F**: Fluorescence intensity of particles in THP-1 cells (treated with LPS or not) treated with particles for 6h, 24h and 72h. (N=3, ns p>0.05, *p<0.05, **p<0.01,***p<0.001)

As was shown in Fig. 4A1-A3, in monocytes without LPS stimulation, the phagocytic effect of monocytes on nanoparticles increased with the prolonged co-incubation time (Fig. 4A1: 6 h; Fig. 4A2: 24 h; Fig. 4A3:72 h), and the differences of fluorescence intensity at different co-incubation times were statistically significant (Fig. 4B). Similarly, monocytes stimulated with LPS would also show the phagocytic effect on nanoparticles with the prolonged co-incubation time (Fig. 4C1: 6 h; Fig. 4C2: 24 h; Fig. 4C3: 72 h). And the result was statistical significance (Fig. 4D). Furthermore, to investigate the targeting mechanism of monocytes towards nanoparticles, we blocked CD14 on LPS-stimulated monocytes using anti-CD14 antibodies and observed a reduction in nanoparticle phagocytosis, indicating that monocyte-targeted phagocytosis of nanoparticles depended on CD14(Figure 4C4), and the differences of fluorescence intensity was statistically significant (Fig. 4D).

To investigate the mechanism of nanoparticles delivering siTET3 into monocytes, monocytes were treated with PMS-fam-siTET3-PEI-PEG/PEI-PEG-CGTArg at a concentration for different durations. Based on the colocalization maps and relevant statistical analyses, it was observed that after 6 h of co-incubation (Fig. 4C1), the nanoparticles (green) and lysosomes (red) largely overlapped. The colocalization map showed a collinearity coefficient of approximately 0.85, indicating that the nanoparticles were captured by the lysosomes. After 24 h of co-incubation (Fig. 4C2), the nanoparticles (green) and lysosomes (red) showed slight overlap, with the colocalization map indicating a coefficient of around 0.4, suggesting reduced fusion between the nanoparticles and lysosomes. After 72 h of co-incubation (Fig. 4C3), the nanoparticles (green) and lysosomes (red) no longer overlapped, and the colocalization map showed no collinearity, indicating that the nanoparticles had been released from the lysosomes, achieving lysosomal escape. Furthermore, additional statistical analyses demonstrated that from 6 h to 72 h of co-incubation, the collinearity between the nanoparticles and lysosomes decreased, confirming lysosomal escape (Fig. 4E).

In order to simulate the inflammatory state of MI, we evaluated the differential phagocytosis of nanoparticles by monocytes with and without LPS stimulation. The study revealed that LPS-stimulated monocytes exhibited enhanced phagocytic activity towards nanoparticles, suggesting that the inflammatory state enhanced monocyte-targeted phagocytosis of nanoparticles (Fig. 4 F).

#### The effect of nanoparticles on monocytes chemotaxis and reprogramming monocytes

**Fig. 5:**
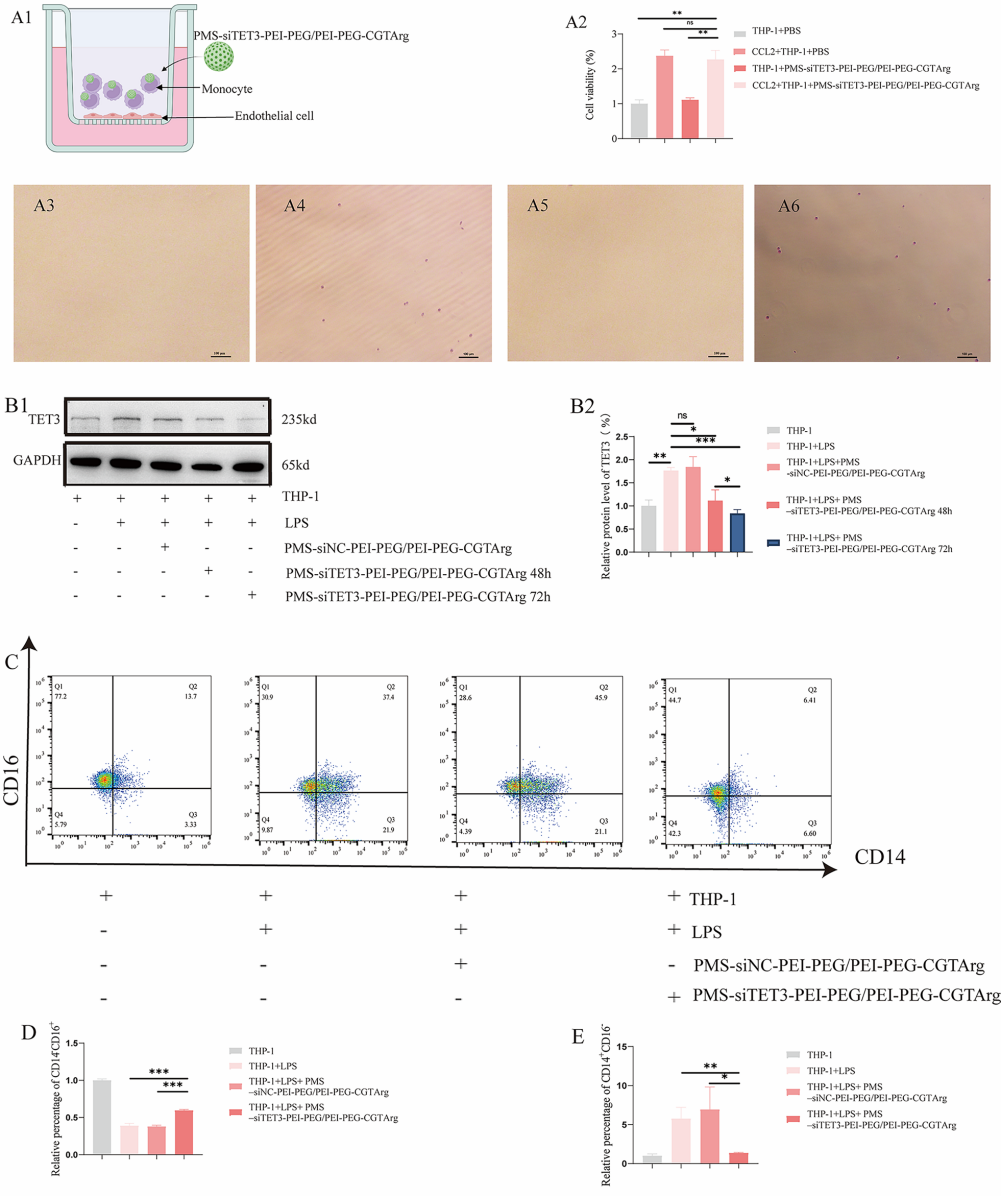
The effects of nanoparticles on monocyte chemotaxis and reprogramming monocytes. **A1**: Schematic diagram of a transwell system; **A2**: Comparison of the effect of different particles on monocyte chemotaxis; **A3**-**A6**: Monocytes in the lower chamber stained with crystal violet (**A3**: THP-1+PBS; A4: CCL2+THP-1+PBS; A5: THP-1+PMS-siTET3-PEI-PEG/PEI-PEG-CGTArg; **A6**: CCL2+THP-1+PMS-siTET3-PEI-PEG/PEI-PEG-CGTArg); WB (**B1**) analysis of monocytes treated with different particles; **B2**: Statistical analysis of the WB results; **C**: Flow cytometry identification of inflammatory monocytes (CD14^+^CD16^-^) and anti-inflammatory monocytes (CD14^-^CD16^+^) treated with different particles; **D**: Comparison of the ratio of CD14^-^CD16^+^ monocytes treated with different particles; **E**: Comparison of the ratio of CD14^+^CD16^-^ monocytes treated with different particles. (N=3, ns p>0.05, *p<0.05,**p<0.01, ***p<0.001)

To investigate the influence of particles on the migration of monocytes, transwell systems were used (Fig. 5A1). Monocytes were treated with particles and transferred to the upper chamber to assess the migration. Chemotaxis of monocytes was not affected with the delivery system, as shown in Fig. 5A2. Furthermore, after staining with crystal violet, monocytes were clearly observed in monocytes treated with particles (Fig. 5A3-A6). This indicated that the nanoparticles did not affect the chemotactic function of monocytes.

According to Fig. 5B1-B2, monocytes treated with LPS showed higher expression of TET3. Additionally, monocytes treated with PMS-siTET3-PEI-PEG/PEI-PEG-CGTArg showed lower expression of TET3 compared to cells treated with PMS-PEI-PEG/PEI-PEG-CGTArg or PMS-siNC-PEI-PEG/PEI-PEG-CGTArg. Moreover, monocytes treated with PMS-siTET3-PEI-PEG/PEI-PEG-CGTArg for 72 h showed lower expression of TET3 compared to monocytes treated PMS-siTET3-PEI-PEG/PEI-PEG-CGTArg for 48 h. This indicated that PMS-siTET3-PEI-PEG/PEI-PEG-CGTArg was successful in knocking down TET3 expression.

Pro-inflammatory monocytes (CD14^+^CD16^−^monocytes) and anti-inflammatory monocytes (CD14^−^CD16^+^ monocytes) were obtained with flow cytometry (Fig. 5 C) in monocytes treated with different particles. Additionally, monocytes treated with PMS-siTET3-PEI-PEG/PEI-PEG-CGTArg for 72 h exhibited a lower proportion of CD14^+^CD16^−^ monocytes and a higher proportion of CD14^−^CD16^+^ monocytes compared to PMS-siNC-PEI-PEG/PEI-PEG-CGTArg (Fig. 5D and E).

### Research on mice

#### Biodistribution and targeting specificity of PMS-siTET3-PEI-PEG/PEI-PEG-CGTArg in vivo

**Fig. 6:**
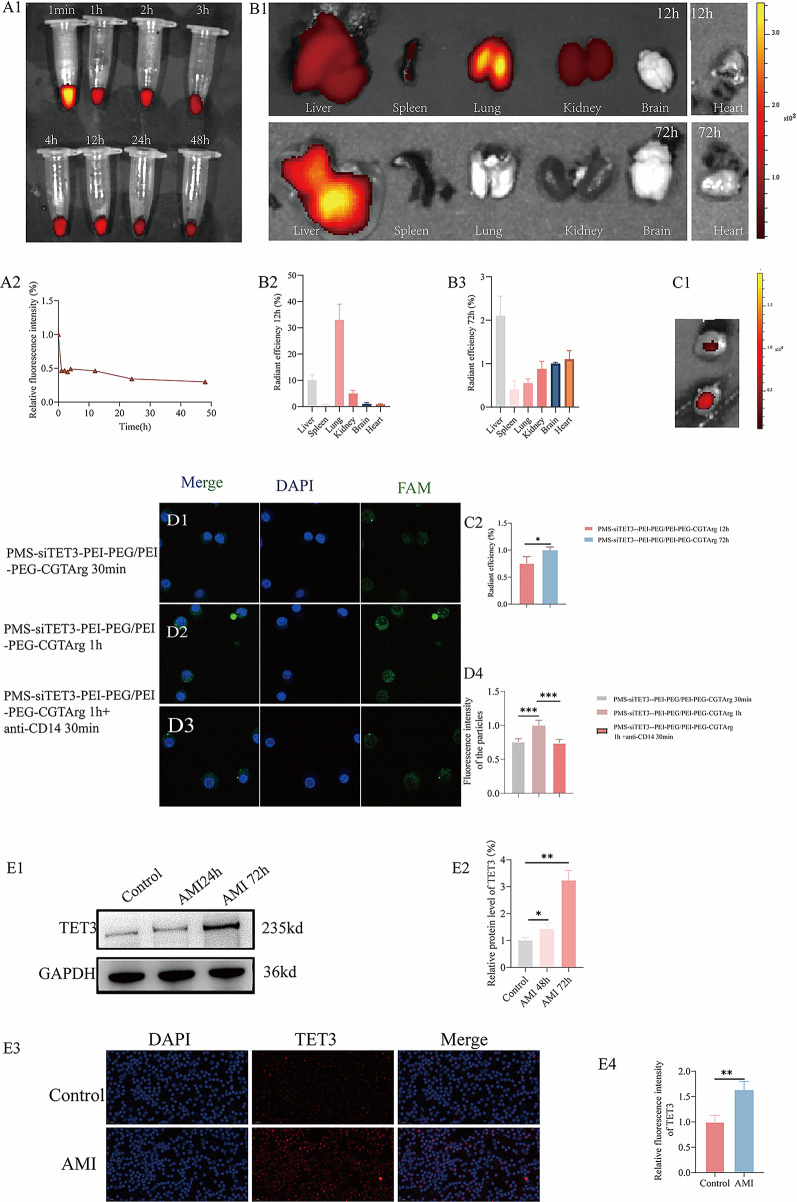
Applications of nanoparticles and TET3 expression in mice. **A1**: IVIS images of PMS-siTET3 (labeled with Cy5)-PEI-PEG/PEI-PEG-CGTArg in mouse blood at different times (1 min, 2 h, 3 h, 4 h, 12 h, 24 h and 48h) post-intravenous injection; **A2**: Relative fluorescence intensity (based on the result of IVIS) of PMS-siTET3 (labeled with Cy5)-PEI-PEG/PEI-PEG-CGTArg in blood at different times post-injection, with fluorescence intensity at 1 min as the reference; **B1**: Representative ex vivo images of major organs (liver, spleen, lung, kidney, brain and heart) at 12 h and 72 h post-intravenous injection of PMS-siTET3 (labeled with Cy5)-PEI-PEG/PEI-PEG-CGTArg; **B2**-**B3**: Relative fluorescence intensity of nanoparticle accumulation in major organs (liver, spleen, brain, lung, kidney and heart) compared to the brain at 12 h (**B2**) and 72 h (**B3**) post-injection; **C1**: Representative ex vivo images of the heart in AMI mice with different treatments, including PMS-siTET3 (labeled with Cy5)-PEI-PEG/PEI-PEG-CGTArg at 12 h and 72 h; **C2**: Relative fluorescence intensity of nanoparticle accumulation in hearts of AMI mice at 12 h compared to 72 h; **D1**-**D3**: Representative images of monocytes in the blood of AMI mice treated with different treatments, including PMS-siTET3 (labeled with FAM)-PEI-PEG/PEI-PEG-CGTArg for 30 min, 1 h, and 1 h (blood was blocked with anti-CD14 for 30 min); **D4**: Relative fluorescence intensity of nanoparticle accumulation in blood monocytes with different treatments, with fluorescence intensity at 1 h as the reference; WB analysis (**E1**) of TET3 expression in monocytes from healthy mice and AMI mice (72h); **E2**: Statistical analysis of the WB data; **E3**: Representative images of monocytes stained with TET3 (red) and DAPI (blue) in healthy mice and AMI mice (72h); E4: Statistical analysis of the differential fluorescence expression. (N=3, ns p>0.05, *p<0.05, **p<0.01,***p<0.001)

To assess the circulation characteristics of the delivery system, PMS-siTET3 (labeled with Cy5)-PEI-PEG/PEI-PEG-CGTArg was intravenously injected into mice. As depicted in the Fig. 6A1, the delivery system could be detected in the blood after tail vein injection. Moreover, relative to the blood concentration at 1 min post-injection, the nanoparticles exhibited a blood circulation within 48 h (Fig. 6A2).

We also evaluated the distribution of the delivery system in organ tissues. As illustrated in Fig. 6B1, 12 h after injection of PMS-siTET3 (labeled with Cy5)-PEI-PEG/PEI-PEG-CGTArg in mice, nanoparticles mainly accumulated in the lungs, liver, and kidneys. After 72 h, the primary accumulation shifted to the liver. With brain fluorescence as a reference group, the similar distribution pattern was detected (Fig. 6B2-B3).

To further assess nanoparticle targeting distribution in AMI mice, two groups of AMI mice were treated with the delivery system. The first group received PMS-siTET3 (labeled with Cy5)-PEI-PEG/PEI-PEG-CGTArg for 12 h, the second group received PMS-siTET3 (labeled with Cy5)-PEI-PEG/PEI-PEG-CGTArg for 72 h. After the treatment, hearts were harvested and subjected to IVIS detection. As was shown in Fig. 6C1, a significant increase in nanoparticle accumulation in the heart at 72 h compared to 12 h. With fluorescence of hearts at 72 h as a reference, the accumulation of particles was consistent (Fig. 6C2).

As shown in the Figure S1D1, we used different flow cytometry antibodies to isolate blood cells, obtaining CD45⁻ cells, CD45⁺CD11b⁻ lymphocytes, CD45⁺CD11b⁺CD115⁺ monocytes, and CD45⁺CD11b⁺CD115⁻Ly6G⁺ neutrophils. The distribution of nanoparticles in different cell populations was then evaluated. The results (Figure S1D2) demonstrated that the nanoparticles were more abundantly distributed in CD45⁺CD11b⁺CD115⁺ monocytes, with a significantly higher level compared to other cell types. In contrast, nanoparticle distribution was considerably lower in CD45⁻ cells, CD45⁺CD11b⁻ lymphocytes, and CD45⁺CD11b⁺CD115⁻Ly6G⁺ neutrophils.

To further explore the targeting mechanism of the nanoparticles, blood of different AMI mice was obtained. The first group was treated with PMS-siTET3 (labeled with fam)-PEI-PEG/PEI-PEG-CGTArg for 30 min (Fig. 6D1), the second group treated with PMS-siTET3 (labeled with fam)-PEI-PEG/PEI-PEG-CGTArg for 1 h (Fig. 6D2). And the third group was blocked with anti-CD14 for 30 min before the blood was treated with PMS-siTET3 (labeled with fam)-PEI-PEG/PEI-PEG-CGTArg for 1 h (Fig. 6D3). As was shown in Fig. 6D4, it was revealed that PMS-siTET3 (labeled with fam)-PEI-PEG/PEI-PEG-CGTArg effectively accumulated in monocytes in the blood with prolonged treatment duration. Additionally, the use of the antibody blocking CD14 resulted in reduced particles accumulation, suggesting that the targeting specificity of PMS-siTET3-PEI-PEG/PEI-PEG-CGTArg relied on CD14.

#### Expression of TET3 in AMI mice

To further assess the expression of TET3 in AMI mice, monocytes were obtained from healthy mice and AMI mice (72 h post-infarction) for WB analysis. The study showed that TET3 was highly expressed in the monocytes of AMI mice (Fig. 6E1-E2). Then, monocytes were also seeded on glass coverslips for immunofluorescence detection. Consistently, TET3 was highly expressed in monocytes of AMI mice (Fig. 6E3-E4).

#### PMS-siTET3-PEI-PEG/PEI-PEG-CGTArg reprograming monocytes in vivo

**Fig. 7:**
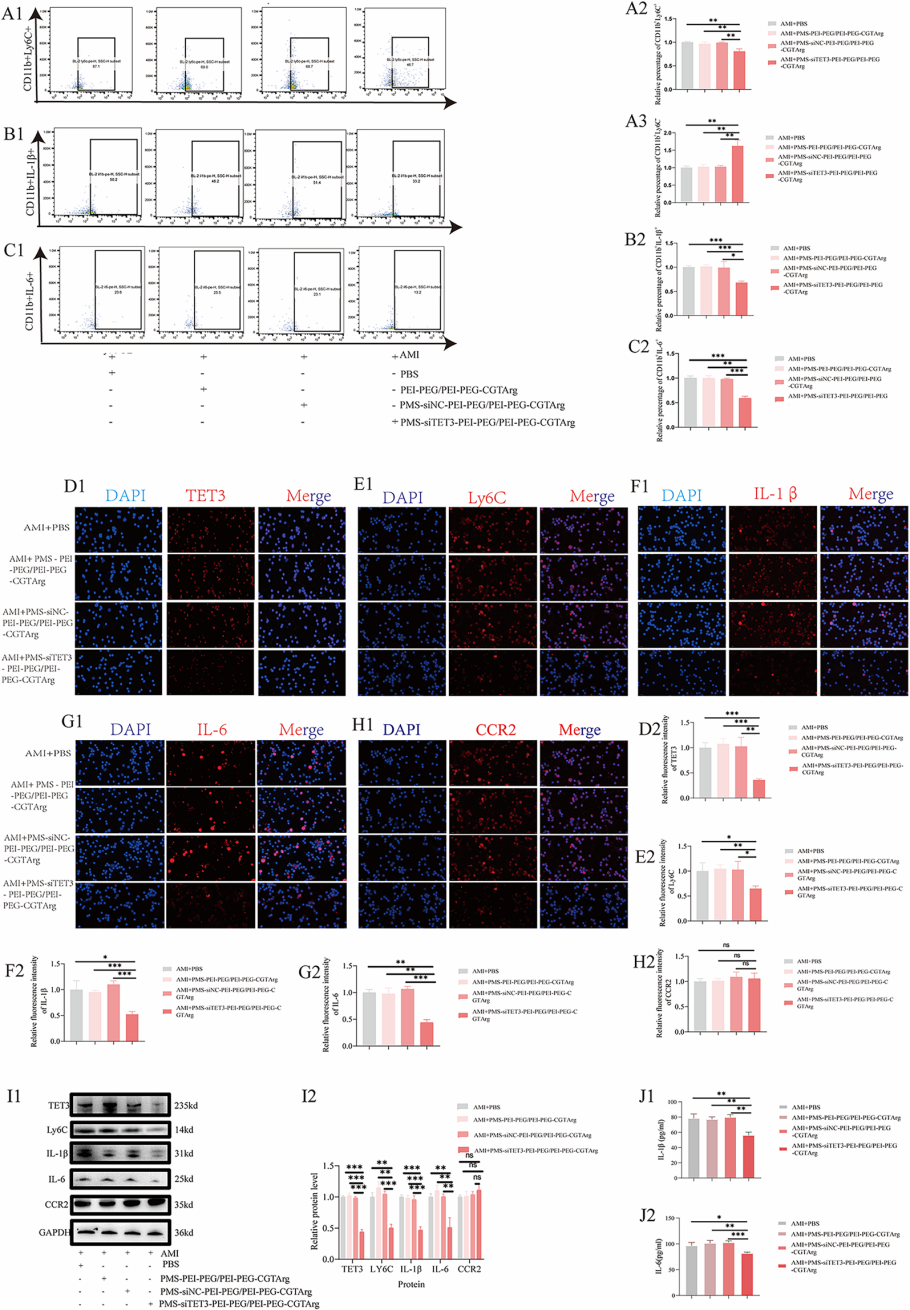
PMS-siTET3-PEI-PEG/PEI-PEG-CGTArg promoted changes between pro-inflammatory monocytes (CD11b^+^Ly6C^+^monocytes) and anti-inflammatory monocytes (CD11b^+^Ly6C^-^monocytes).**A1**: Representative flow cytometry plots of pro-inflammatory monocytes (CD11b^+^Ly6C^+^ monocytes) and anti-inflammatory monocytes (CD11b^+^Ly6C^-^ monocytes) in the blood of AMI mice treated with PBS, PMS-PEI-PEG/PEI-PEG-CGTArg, PMS-siNC-PEI-PEG/PEI-PEG-CGTArg and PMS-siTET3-PEI-PEG/PEI-PEG-CGTArg; The percentage statistical analysis of pro-inflammatory monocytes (CD11b^+^Ly6C^+^ monocytes) (**A2**) and anti-inflammatory monocytes (CD11b^+^Ly6C^-^ monocytes) (**A3**); Representative flow cytometry plots of CD11b^+^ IL-1β^+^ monocytes (**B1**) and CD11b^+^ IL-6^+^ monocytes (**C1**) in the blood of AMI mice treated with PBS, PMS-PEI-PEG/PEI-PEG-CGTArg, PMS-siNC-PEI-PEG/PEI-PEG-CGTArg and PMS-siTET3-PEI-PEG/PEI-PEG-CGTArg; The percentage statistical analysis of CD11b^+^ IL-1β^+^ monocytes (**B2**) and CD11b^+^IL-6^+^ monocytes (**C2**); Representative fluorescence images of monocytes (**D1**: TET3; **E1**: Ly6C; **F1**: IL-1β; **G1**: IL-6; **H1**: CCR2) in AMI mice treated with PBS, PMS-PEI-PEG/PEI-PEG-CGTArg, PMS-siNC-PEI-PEG/PEI-PEG-CGTArg, PMS-siTET3-PEI-PEG/PEI-PEG-CGTArg; Statistical analysis of the fluorescence expression of different markers (**D2**: TET3; **E2**: Ly6C; **F2**: IL-1β; **G2**: IL-6; **H2**: CCR2) in monocytes. WB (**I1**) analysis of the effect of different nanoparticles (including PBS, PMS-PEI-PEG/PEI-PEG-CGTArg, PMS-siNC-PEI-PEG/PEI-PEG-CGTArg, PMS-siTET3-PEI-PEG/PEI-PEG-CGTArg) on the expression of TET3, Ly6C, IL-1β, IL-6 and CCR2 in monocytes; **I2**: Statistical analysis of the WB data; Comparison on the concentration of serum IL-1β (**J1**) and IL-6 (**J2**) in AMI mice treated with PBS, PMS-PEI-PEG/PEI-PEG-CGTArg, PMS-siNC-PEI-PEG/PEI-PEG-CGTArg or PMS-siTET3-PEI-PEG/PEI-PEG-CGTArg. (N=3，ns p>0.05, *p<0.05, **p<0.01,***p<0.001)

Representative flow cytometry plots of pro-inflammatory monocytes (CD11b^+^Ly6C^+^monocytes) and anti-inflammatory monocytes (CD11b^+^Ly6C^−^ monocytes) were shown in Fig. 7A1 in the blood of AMI mice treated with PBS, PMS-PEI-PEG/PEI-PEG-CGTArg, PMS-siNC-PEI-PEG/PEI-PEG-CGTArg and PMSsiTET3-PEI-PEG/PEI-PEG-CGTArg. Our study revealed that PMS-siTET3-PEI-PEG/PEI-PEG-CGTArg effectively reduced the proportion of inflammatory monocytes (CD11b^+^Ly6C^+^ monocytes, Fig. 7A2) and increased the proportion of anti-inflammatory monocytes (CD11b^+^Ly6C^−^ monocytes, Fig. 7A3).

Representative flow cytometry plots of CD11b^+^IL-1β^+^ monocytes (Fig. 7B1) and CD11b^+^IL-6^+^ monocytes (Fig. 7C1) were obtained in the AMI mice treated with PBS, PMS-PEI-PEG/PEI-PEG-CGTArg, PMS-siNC-PEI-PEG/PEI-PEG-CGTArg and PMSsiTET3-PEI-PEG/PEI-PEG-CGTArg. Similarly, it was shown that PMS-siTET3-PEI-PEG/PEI-PEG-CGTArg effectively reduced the proportion of CD11b^+^IL-1β^+^ monocytes (Fig. 7B2) and CD11b^+^IL-6^+^ monocytes (Fig. 7C2).

Furthermore, monocytes were isolated and seeded onto cell slides. Then, immunofluorescence was employed to assess the impact of PMS-siTET3-PEI-PEG/PEI-PEG-CGTArg on the levels of inflammatory markers in monocytes. The study demonstrated that PMS-siTET3-PEI-PEG/PEI-PEG-CGTArg effectively reduced TET3 expression in monocytes (Fig. 7D1 and Fig. 7D2). In addition, PMS-siTET3-PEI-PEG/PEI-PEG-CGTArg effectively reduced the expression of inflammatory markers, including Ly6C (Fig. 7E1 and Fig. 7E2), IL-1β (Fig. 7F1 and Fig. 7F2) and IL-6 (Fig. 7G1 and Fig. 7G2).

Similarly, AMI mice were treated with PBS, PMS-PEI-PEG/PEI-PEG-CGTArg, PMS-siNC-PEI-PEG/PEI-PEG-CGTArg, and PMS-siTET3-PEI-PEG/PEI-PEG-CGTArg. Three days after the treatment, monocytes were isolated from AMI mice for WB analysis. The study demonstrated that PMS-siTET3-PEI-PEG/PEI-PEG-CGTArg effectively reduced TET3 expression in monocytes. Moreover, regarding inflammatory markers, PMS-siTET3-PEI-PEG/PEI-PEG-CGTArg effectively decreased the levels of Ly6C, IL-1β, and IL-6 (Fig. 7I1-I2). Moreover, PMS-siTET3-PEI-PEG/PEI-PEG-CGTArg would reduce the concentration of IL-1β (Fig. 7J1) and IL-6 (Fig. 7J2) in serum of AMI mice.

In summary, PMS-siTET3-PEI-PEG/PEI-PEG-CGTArg effectively reduced the proportion of inflammatory monocytes, increased the proportion of anti-inflammatory monocytes.

#### Effect of PMS-siTET3-PEI- PEG/PEI-PEG-CGTArg on chemotaxis of monocytes

Considering the inflammatory state, monocytes relied on CCR2 for chemotaxis into the infarcted area. We further evaluated the influence of the delivery system on the expression of CCR2 in monocytes.

As was shown in Fig. 7H1 and Fig. 7H2, Fig. 7I1-I2, PMS-siTET3-PEI-PEG/PEI-PEG-CGTArg did not significantly alter the expression of CCR2 in monocytes. Meaningfully, PMS-siTET3-PEI-PEG/PEI-PEG-CGTArg would not influence the chemotaxis of monocytes into myocardium.

#### Effect of PMS-siTET3-PEI- PEG/PEI-PEG-CGTArg on the distribution of monocytes/macrophages

After three days of treatment with PBS, PMS-PEI-PEG/PEI-PEG-CGTArg, PMS-siNC-PEI-PEG/PEI-PEG-CGTArg, and PMS-siTET3-PEI-PEG/PEI-PEG-CGTArg, the study demonstrated that PMS-siTET3-PEI-PEG/PEI-PEG-CGTArg effectively reduced the infiltration of monocytes/macrophages in the infarct area(Figure S2A1).

#### Cardiac protection of PMS-siTET3-PEI-PEG/PEI-PEG-CGTArg

**Fig. 8:**
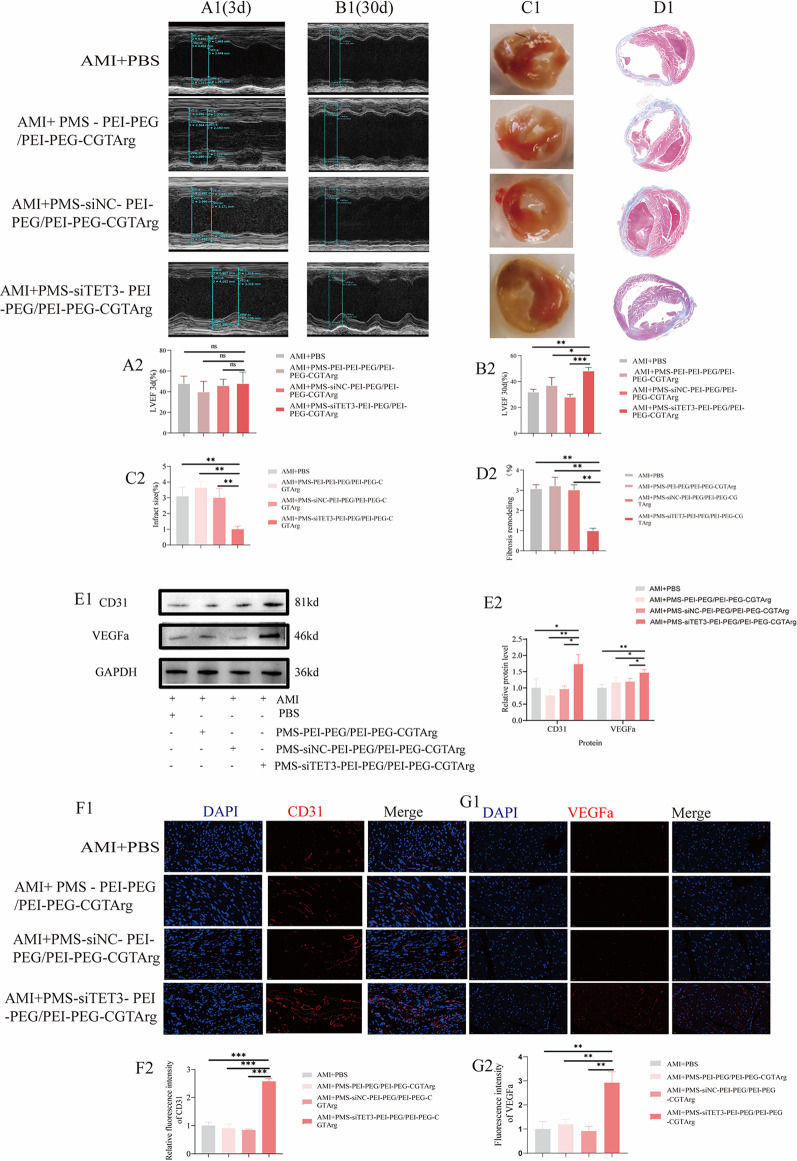
Cardiac protection of PMS-siTET3-PEI-PEG/PEI-PEG-CGTArg in mice. Cardiac function was evaluated via echocardiography at 3 days (**A1**) and 30 days (**B1**) after treatment with different particles (PBS, PMS-PEI-PEG/PEI-PEG-CGTArg, PMS-siNC-PEI-PEG/PEI-PEG-CGTArg, PMS-siTET3-PEI-PEG/PEI-PEG-CGTArg). Statistical analysis of LVEF in AMI mice at 3 days (**A2**) and 30 days (**B2**) after treatment with different particles; **C1**: TTC staining of heart sections in AMI mice at 3 days after treatments; **C2**: Statistical analysis of heart infarct size in AMI mice treated with different particles;**D1**: Masson staining of heart sections in AMI mice at 30 days after treatments;**D2**: Statistical analysis of heart fibrosis area in AMI mice treated with different particles; WB (**E1**) analysis of the effect of different nanoparticles (PBS, PMS-PEI-PEG/PEI-PEG-CGTArg, PMS-siNC-PEI-PEG/PEI-PEG-CGTArg, PMS-siTET3-PEI-PEG/PEI-PEG-CGTArg) on the expression of CD31 and VEGFa in myocardial tissue of AMI mice; **E2**: Statistical analysis of the WB data; Representative fluorescence images of CD31 (**F1**) and VEGFa (**G1**) in AMI mice treated with PBS, PMS-PEI-PEG/PEI-PEG-CGTArg, PMS-siNC-PEI-PEG/PEI-PEG-CGTArg, PMS-siTET3-PEI-PEG/PEI-PEG-CGTArg; Statistical analysis of the differential fluorescence expression of CD31 (**F2**) and VEGFa (**G2**). (N=3, ns p>0.05,*p<0.05, **p<0.01, ***p<0.001)

AMI mice were subjected to different treatment, including PBS, PMS-PEI-PEG/PEI-PEG-CGTArg, PMS-siNC-PEI-PEG/PEI-PEG-CGTArg and PMS-siTET3-PEI-PEG/PEI-PEG-CGTArg. And cardiac ultrasound examinations were conducted 3 days and 30 days after the treatment. The research demonstrated that PMS-siTET3-PEI-PEG/PEI-PEG-CGTArg effectively improved LVEF 30 days after the treatment (Fig. 8B1 and Fig. 8B2). In contrast, no significant differences in LVEF were observed among the four groups after three days of treatment (Fig. 8A1 and Fig. 8A2).

Similarly, AMI mice underwent the four treatments. Three days after the treatment, mouse cardiac tissues were obtained for TTC staining. The study revealed that, compared to the other groups, PMS-siTET3-PEI-PEG/PEI-PEG-CGTArg significantly reduced the infarct area (Fig. 8C1 and Fig. 8C2). Moreover, AMI mice undergoing the four treatments had their cardiac tissues collected for Masson staining one-month post-treatment to assess myocardial fibrosis. The study showed that, compared to the other groups, PMS-siTET3-PEI-PEG/PEI-PEG-CGTArg inhibited myocardial fibrosis (Fig. 8D1 and Fig. 8D2).

Furthermore, we evaluated the impact of the nanoparticles on border zone cardiomyocytes. The study found that in mice treated with PMS-siTET3-PEI-PEG/PEI-PEG-CGTArg, the border zone cardiomyocytes exhibited relatively regular morphology and orderly arrangement. In contrast, the surviving myocardial fibers in the border zone of mice in other groups showed loose arrangement, disorganized orientation, and exhibited significant wavy distortion or fragmentation (Figure S2B).

In addition, one month after the treatment, cardiac tissues were extracted and subjected for WB analysis. The results demonstrated that PMS-siTET3-PEI-PEG/PEI-PEG-CGTArg effectively increased the expression of CD31 and VEGFa (Fig. 8E1-E2). Furthermore, cardiac tissues were also extracted and subjected to immunofluorescence detection. The research revealed that, compared to the other groups, PMS-siTET3-PEI-PEG/PEI-PEG-CGTArg effectively increased the fluorescence density of CD31 (Fig. 8F1 and Fig. 8F2) and VEGFa (Fig. 8G1 and Fig. 8G2), contributing to angiogenesis in cardiac tissues.

#### Validation of overexpression following nanoparticle therapy

The study showed that TET3 could still be induced to express even after its initial downregulation (Figure S3A1–A2). Furthermore, this induction led to an increase in pro-inflammatory monocytes (Figure S3B1–B2).

#### Mechanism of tet3 in regulating the Notch signaling pathway in monocytes

Transcriptomic sequencing revealed that PMS-siTET3-PEI-PEG/PEI-PEG-CGTArg led to decreased expression of Notch1, HES1, and DTX2, but had little effect on DLL4 (Figure S1C1). Further validation by PCR showed that while PMS-siTET3-PEI-PEG/PEI-PEG-CGTArg had no significant effect on Notch1 expression, it significantly reduced the expression of HES1 and DTX2, with statistically significant differences (Figure S3C1-C2).

#### Off-target effects of siTET3

Based on sequencing and bioinformatic analysis, six off-target genes were identified: Rtp4, Snn, Phf11a, Eva1b, Ifit3, and Ier5l. These were further validated using the newly designed siTET3 sequences. The results showed that the new siTET3 sequences did not induce significant changes in the expression of these six off-target genes, including Rtp4, Snn, Phf11a, Eva1b, Ifit3, and Ier5l (Figure S3D1-D2).

#### Safety of PMS-siTET3-PEI-PEG/PEI-PEG-CGTArg in mice

To further assess the safety of nanoparticles in mice, the mice were intravenously injected with PMS-siTET3-PEI-PEG/PEI-PEG-CGTArg. Blood samples were collected at different time points post-injection, and various hematological parameters were analyzed. The study revealed that within 1 week of tail vein injection, PMS-siTET3-PEI-PEG/PEI-PEG-CGTArg did not cause significant abnormalities in blood parameters, indicating a lack of apparent toxicity (Figure S4A1-A10).

Furthermore, mice were divided into four groups: one receiving PBS treatment, the second group treated with PMS-siTET3-PEI-PEG/PEI-PEG-CGTArg for one week, the third group was treated with PMS-siTET3-PEI-PEG/PEI-PEG-CGTArg for one month and the fourth group was treated with PMS-siTET3-PEI-PEG/PEI-PEG-CGTArg for two times during one month, the fifth group was treated with PMS-siTET3-PEI-PEG/PEI-PEG-CGTArg for three months. Subsequently, major organs including the liver, spleen, lungs, kidneys and brain were collected and subjected to HE staining. The study demonstrated that important organ tissues did not exhibit noticeable pathological changes, suggesting that PMS-siTET3-PEI-PEG/PEI-PEG-CGTArg did not display apparent organ toxicity during the treatment period (Figure S4B1-B5). Our study demonstrated that, compared to the control group, the nanoparticles did not induce significant production of specific antibodies after one month of treatment.

### Research on pigs

#### AMI model

**Fig. 9:**
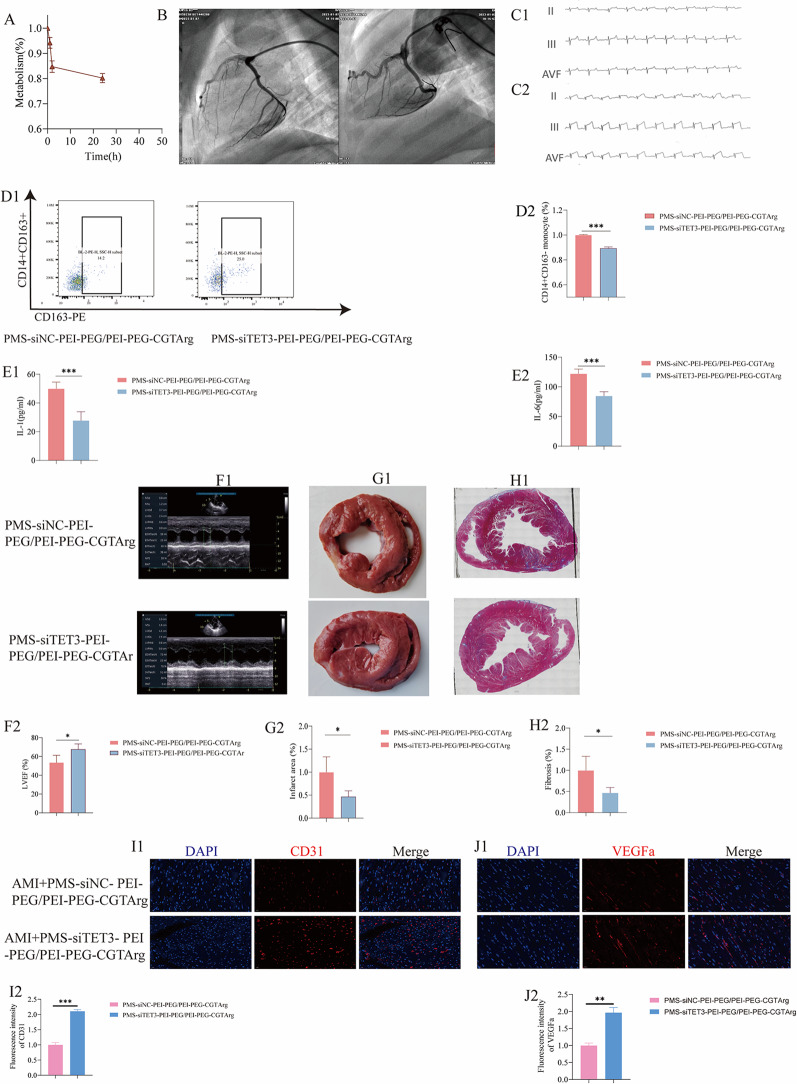
PMS-siTET3-PEI-PEG/PEI-PEG-CGTArg application in the treatment of AMI pigs. **A**: Metabolism of PMS-siTET3-PEI-PEG/PEI-PEG-CGTArg in blood based on the relative abundance of silicon element; **B**: CAG of coronary artery; **C1**: ECG before occlusion of coronary artery; **C2**: ECG after occlusion of coronary artery; **D1**: Representative flow cytometry plots of pro-inflammatory monocytes (CD14^+^CD163^-^monocytes) in the blood of AMI pigs treated with PMS-siNC-PEI-PEG/PEI-PEG-CGTArg or PMS-siTET3-PEI-PEG/PEI-PEG-CGTArg; **D2**: Percentage statistical analysis of pro-inflammatory monocytes; ELISA analysis of IL-1 (**E1**) and IL-6 (**E2**) concentrations in blood of AMI pigs after treatment with PMS-siNC-PEI-PEG/PEI-PEG-CGTArg or PMS-siTET3-PEI-PEG/PEI-PEG-CGTArg; **F1**: Echocardiography revealed cardiac function in pigs treated with PMS-siNC-PEI-PEG/PEI-PEG-CGTArg or PMS-siTET3-PEI-PEG/PEI-PEG-CGTArg; **F2**: Comparison of LVEF in pigs treated with PMS-siNC-PEI-PEG/PEI-PEG-CGTArg or PMS-siTET3-PEI-PEG/PEI-PEG-CGTArg; **G1**: TTC staining of heart sections from pigs treated with PMS-siNC-PEI-PEG/PEI-PEG-CGTArg or PMS-siTET3-PEI-PEG/PEI-PEG-CGTArg; **G2**: Statistical analysis of heart infarct size in AMI pigs treated with different particles; **H1**: Masson staining of heart sections from pigs treated with PMS-siNC-PEI-PEG/PEI-PEG-CGTArg or PMS-siTET3-PEI-PEG/PEI-PEG-CGTArg; **H2**: Statistical analysis of heart fibrosis area in AMI pigs treated with different particles; Representative fluorescence images of CD31 (**I1**) and VEGFa (**J1**) in AMI pigs treated with PMS-siNC-PEI-PEG/PEI-PEG-CGTArg or PMS-siTET3-PEI-PEG/PEI-PEG-CGTArg; Statistical analysis of the differential fluorescence expression of CD31 (**I2**) and VEGFa (**J2**). (N=4, ns p>0.05, *p<0.05, **p<0.01, ***p<0.001)

To investigate the in vivo metabolism of the delivery system in pigs, the presence of PMS-siTET3-PEI-PEG/PEI-PEG-CGTArg in the bloodstream was examined. Following intravenous injection via the ear vein, as depicted in the Fig. 9 A, the delivery system demonstrated favorable circulation and maintained a detectable drug concentration in the blood.

As depicted in Fig. 9B, Coronary angiography (CAG) was displayed. And Fig. 9C1 represented the electrocardiograph (ECG) before occlusion of the coronary artery. Following the successful balloon occlusion of the coronary artery, Fig. 9C2 illustrated a substantial elevation in the ST segment on the ECG. These observations strongly indicated the successful establishment of an AMI model in pigs.

#### PMS-siTET3-PEI-PEG/PEI-PEG-CGTArg reprograming monocytes in AMI pigs

To further investigate the effects of the delivery system on inflammation, PBMCs were isolated from AMI pigs three days post-treatment and subjected to flow cytometry analysis. Representative flow cytometry plots of pro-inflammatory monocytes (CD14^+^CD163^−^ monocytes) were shown in Fig. 9D1 in the AMI pigs treated with PMS-siNC-PEI-PEG/PEI-PEG-CGTArg and PMS-siTET3-PEI-PEG/PEI-PEG-CGTArg. Compared to PMS-siNC-PEI-PEG/PEI-PEG-CGTArg, PMS-siTET3-PEI-PEG/PEI-PEG-CGTArg significantly reduced pro-inflammatory monocytes (CD14^+^CD163^−^ monocytes) (Fig. 9D2) in AMI pigs.

Furthermore, it was demonstrated that the PMS-siTET3-PEI-PEG/PEI-PEG-CGTArg significantly decreased the concentrations of inflammatory cytokines in blood, including IL-1 (Fig. 9E1) and IL-6 (Fig. 9E2).

#### Cardiac protection of the delivery system

One month after AMI pigs injected with the delivery system, echocardiography was performed to assess cardiac function. The results of echocardiography were presented for pigs treated with PMS-siNC-PEI-PEG/PEI-PEG-CGTArg and PMS-siTET3-PEI-PEG/PEI-PEG-CGTArg. Compared to the pigs treated with PMS-siNC-PEI-PEG/PEI-PEG-CGTArg, pigs treated with PMS-siTET3-PEI-PEG/PEI-PEG-CGTArg were associated with improved cardiac function, as indicated by the increased LVEF (Fig. 9F1 and Fig. 9F2) (*p* < 0.05).

Subsequently, to further assess the impact of the delivery system on myocardial infarction size and myocardial fibrosis in pigs, tissue sections were prepared and subjected to TTC staining and masson’s staining. In comparison to PMS-siNC-PEI-PEG/PEI-PEG-CGTArg, pigs treated with PMS-siTET3-PEI-PEG/PEI-PEG-CGTArg displayed smaller areas of myocardial infarction size (Fig. 9G1 and Fig. 9G2) and significantly reduced myocardial fibrosis (Fig. 9H1 and Fig. 9H2).

Furthermore, cardiac sections were also extracted and subjected to immunofluorescence detection. The research revealed that PMS-siTET3-PEI-PEG/PEI-PEG-CGTArg compared to PMS-siNC-PEI-PEG/PEI-PEG-CGTArg effectively increased the fluorescence density of CD31 (Fig. 9I1 and Fig. 9I2) and VEGFa (Fig. 9J1 and Fig. 9J2).

We further evaluated the effect of the nanoparticles on vessel density. According to the magnification and scale bar of the fluorescence microscopy method used, the area of the acquired field of view was calculated as: 0.155 mm × 0.304 mm = 0.047 mm². Tissue vessel density was assessed based on CD31 staining, and was determined as the number of CD31-positive cells per 0.047 mm². Our study showed that the vessel density in the infarcted area of AMI pigs treated with PMS-siTET3-PEI-PEG/PEI-PEG-CGTArg was approximately 1553/mm². In comparison, the vessel density in the infarcted area of AMI pigs treated with PMS-siNC-PEI-PEG/PEI-PEG-CGTArg was approximately 2361/mm².

#### Safety of PMS-siTET3-PEI-PEG/PEI-PEG-CGTArg in pigs

In healthy pigs, a series of safety-related experiments were conducted involving intravenous injection of NS or the delivery system. Peripheral blood samples were collected at different time points after injection. As shown in Figure S5A1-A6, the results indicated that the delivery system did not demonstrate significant hepatotoxicity, nephrotoxicity and inflammation.

Regarding major organs collected one month after delivery system injection, no significant pathological changes were observed (Figure S5B1). Additionally, there were no noticeable differences in organ weights between the three groups, including the liver, spleen, lung, and kidney (Figure S5B2-B5). Moreover, histopathological examination of tissue sections did not reveal significant pathological alterations compared to the pig treated with NS (Figure S5C1-C3).

## Discussion

Based on the PMS platform, we developed a novel delivery system capable of efficiently encapsulating siTET3 with high stability and biosafety. This system enabled effective intracellular delivery of siTET3 into monocytes, leading to monocyte reprogramming and a significant reduction in inflammatory responses. As a result, the therapeutic intervention contributed to improved outcomes in acute myocardial infarction (AMI). Notably, we further validated the anti-inflammatory efficacy and prognostic benefits of the PMS-siTET3-PEI-PEG/PEI-PEG-CGTArg complex in a large animal model of AMI.

After MI, necrotic cells released danger signals, known as Damage-Associated Molecular Patterns (DAMPs). DAMPs bound to pattern recognition receptors (PRRs) on infiltrating leukocytes of the innate immune system, thereby strongly activating a cascade of inflammatory mediators, including cytokines, chemokines, and cell adhesion molecules, which ultimately exacerbated the inflammatory response in the infarcted area [19]. Yang et al. utilized nuclear magnetic resonance imaging (MRI) to localize myocardial inflammation and edema using T2 mapping. The study found that compared to healthy pigs, remote T2 significantly increased from 3 days to 3 months post-MI (31.43 ± 0.67 vs. 33.53 ± 1.15 vs. 36.43 ± 1.07 msec), indicating the inflammation post-MI. Similarly, compared to healthy patients, MI patients exhibited a significant increase in T2 (40.51 ± 1.79 vs. 41.94 ± 1.14 vs. 42.52 ± 1.71 milliseconds). Furthermore, in MI patients, remote T2 at 3 months (β = 0.432) and the variability of remote T2 between the two scans (β = 0.554) were independently associated with left ventricular remodeling [20]. Based on this, studies have focused on anti-inflammatory therapy for AMI. Wang et al. synthesized a colchicine delivery system based on calcium carbonate nanoparticles (ColCaNPs) using the broad-spectrum anti-inflammatory drug, colchicine. Subsequently, they conducted drug intervention therapy on AMI mice. The research demonstrated that the colchicine delivery system encapsulated in nanoparticles effectively reduced the levels of serum C-reactive protein (CRP), tumor necrosis Factor-alpha (TNF-α), and IL-1β, decreased the infarct area by 45%, and alleviated myocardial fibrosis [21].

Produced in the bone marrow and spleen, monocytes were recruited to injured myocardium in the two phases described above, during which Ly6c^high^ monocytes caused the expanding of infarcted area and left ventricle (LV) remodeling, whereas Ly-6c^low^ monocytes inhibiting scar formation [7, 22]. In an observational study, Bosch et al. evaluated the correlation between inflammatory monocytes (CD14^+^CD16^−^ monocytes) and cardiac scar formation in ST-segment elevation myocardial infarction (STEMI) patients. The study included 102 patients with first-diagnosed STEMI. The results showed that peak levels of inflammatory monocytes were negatively correlated with LVEF at 7 days (*r* = −0.30, *p* = 0.003) and 6 months (*r* = −0.31, *p* = 0.002) in STEMI patients. Additionally, elevated CD14^+^CD16^−^ monocytes were strongly associated with myocardial scars [23]. These findings suggested that increased levels of inflammatory monocytes contributed to myocardial remodeling and the poor prognosis of AMI patients.

In our study, PMS-siTET3-PEI-PEG/PEI-PEG-CGTArg was able to reduce the levels of pro-inflammatory monocytes and increase the levels of anti-inflammatory monocytes. The reduction in pro-inflammatory monocytes led, on one hand, to a decrease in pro-inflammatory cytokines such as IL-1 and IL-6, which in turn reduced myocardial fibrosis, which would ultimately contribute to reduced myocardial fibrosis and improved cardiac function.

Similar to our study, previous research has aimed to improve AMI prognosis by intervening with pro-inflammatory monocytes and anti-inflammatory monocytes. It was shown that inhibiting inflammatory monocytes recruitment to the infarcted myocardium would significantly reduce the infarct size and improve the prognosis of myocardial infarction [24, 25], which made a demonstration that decreasing the inflammatory monocytes would improve the prognosis of AMI.

In comparison, interventions targeting tissue macrophages to achieve a reprogramming of macrophage proportions, improving the prognosis of AMI. One study demonstrated that PMS could efficiently deliver miRNA-21 to macrophages, thereby reprogramming inflammatory macrophages into anti-inflammatory macrophages, which effectively protected the cardiac function of AMI mice. Additionally, Tan et al. found that the designed nanoparticles exhibited higher transfection efficiency in pro-inflammatory macrophages. Moreover, fibrosis staining of mouse myocardial tissue revealed that the fibrotic area before treatment was approximately three times larger than that after treatment, which is consistent with our research findings [17]. In addition, more studies were focused on macrophages, while our study was concentrated on monocytes [26, 27]. In the early stages of inflammation, monocytes play a crucial role. Compared to macrophages, targeting monocytes was more conducive to early inflammation intervention and thus improving the prognosis of AMI.

Considering that monocytes have a weaker phagocytic effect than macrophages, siRNA delivery was more challenging. Moreover, there were various difficulties in siRNA delivery, including siRNA instability and interference from lysosomes. Currently, membrane fusion and the proton sponge effect were the two primary mechanisms involved in the successful delivery of siRNA. In our study, the proton sponge effect was applied using PEI to deliver the siRNA [15, 16]. Corresponding research also indicated that relying solely on the membrane fusion mechanism, although achieving targeted contact with monocytes, makes it challenging to successfully deliver siRNA into monocytes [17]. Therefore, in this study, we relied on the proton sponge effect formed by PEI and the appropriate combination of PEG to achieve targeted delivery of siRNA.

Previous studies have demonstrated that peptides rich in arginine enhance membrane penetration capability, and the addition of tryptophan enhanced their ability to enter cells. Based on this, a combination of arginine [28], tryptophan [29], and PEG was utilized in our study, achieving targeted delivery to monocytes. Research has shown that nanoparticles rely on CD14 for targeted delivery to monocytes in both mouse and human blood. In addition, nanoparticles were significantly less likely to enter other cells in the bloodstream, highlighting the targeted characteristics [18]. Similarly, in our study, we employed arginine, tryptophan, and PEG for rational modification, completing the encapsulation of PMS complexes, facilitating targeted delivery and membrane penetration to monocytes. Blocking CD14 with anti-CD14 reduced the targeting efficiency. This suggested that our nanomaterials relied on binding to CD14 on monocytes to achieve targeted contact.

Furthermore, we have taken measures in our study to improve transfection efficiency, including the change of the surface potential of particles. As the negative charge on the surface of particles would negatively affect the phagocytosis of the delivery system, PEI was applied to the surface of the PMS [30]. According to our findings, when PEI applied to the surface, the potential became positive, which would facilitate the interaction between the delivery system and monocytes. In addition, PEG and CGTArg was also used in our study, which would help the particles enter the cells [31, 32].

Current research suggested that TET3, as a methyltransferase, primarily regulated cell differentiation. Importantly, existing studies have found that TET3 played a crucial role in the occurrence [33] and development of inflammation [34]. Regarding the potential mechanisms by which TET3 could promote inflammatory monocytes transformation into anti-inflammatory monocytes, it has been suggested that monocytes may be regulated by lineage-specific promoters, and hypermethylation has been associated with a decrease in intermediate and nonclassical monocyte fractions in the blood. In addition, Notch signaling might also play an important role during the process [9]. In the study on the role of TET3 in steroid-associated osteonecrosis, Zhao et al. found that TET3 influenced multiple cellular pathways by inducing alterations in 5hmC methylation. Further sequencing analysis revealed changes in the mRNA expression levels of Notch2, Notch4, Dkk1, and Dkk4 [35]. Gonzalez et al. investigated the protective effect of TET3-mediated 5hmC formation on intestinal epithelial integrity under pathogen infection and chemical stress conditions, and evaluated its impact on the Notch pathway. They similarly demonstrated that TET3 functioned by modulating 5hmC methylation and influenced Notch signaling [36]. Another study uncovered the critical role of TET3-mediated DNA hydroxymethylation of ZMIZ1, a mechanism that activates the Notch1/c-Myc axis, alters macrophage polarization status, and ultimately affects hepatocellular carcinoma progression. By transfecting Kupffer cells (KCs) with sh-TET3, the authors observed that TET3 knockdown led to a significant increase in methylation levels within the ZMIZ1 promoter region. Moreover, the expression of classical Notch1 target genes—including HES1, HES4, and DTX2—was downregulated in TET3-silenced KCs [37]. Similarly, our study also revealed analogous findings, demonstrating that reduced TET3 expression led to alterations in the Notch signaling pathway. To further evaluate the mechanism of TET3, Liu et al. investigated the epigenetic role of TET3 in macrophages and found that it mediated downstream gene transcription in a STAT3-dependent manner by regulating DNA methylation status, thereby further influencing the expression levels of genes such as TGFβ1, NLRP3, and IL-1β [38]. 

Additionally, with the potential value of the delivery system for future clinical research, we further evaluated the safety of the system in our study. PEI was commonly used for gene delivery, including our study, but it exhibited strong cytotoxicity. The reasons included: the large molecular weight and the presence of bulky groups. Therefore, it was of great significance to further reduce the toxicity of PEI in research. Polymers such as PEG and cell-penetrating peptides were commonly used solutions. Cell-penetrating peptides typically contained positively charged amino acid residues, such as arginine and lysine. Commonly used penetrating peptides included KALA, among others [39–42]. Yan et al. further reduced the cytotoxicity of PEI by using the cell-penetrating peptide KALA. Yan et al. developed the nanoparticle system MSN-miR-26@PEI-KALA and evaluated the application in mesenchymal liver cells. The study found that after KALA modification, MSN-miR-26@PEI-KALA did not exhibit significant cytotoxicity [43]. In our research, we designed and synthesized a novel cell-penetrating peptide, CGTArg, and constructed the nanoparticle system PMS-siTET3-PEI-PEG/PEI-PEG-CGTArg, which exhibited low cytotoxicity. According to our study, once modified with CGTArg, drug injection did not cause hemolysis of RBCs in the blood, and further investigation in hepatocytes suggested that it would not cause significant liver damage, especially at low concentrations.

## Methods and statistical analysis

Methods and statistical analysis were available in the Supplementary Information.

## Limitation

In our study, although we used large animals to further verify the therapeutic effects of nanoparticles, this validation was relatively simple. More comprehensive data will be needed before proceeding to clinical studies.

## Conclusion

In our study, we designed and developed a delivery system utilizing PMS as the delivery platform for loading siTET3. This system achieved targeting and entry into monocytes via the CD14 receptor, thereby altering the inflammatory phenotype of monocytes and improving the prognosis of AMI.

## Supplementary Information

Supplementary Material 1.

Supplementary Material 2.

## Data Availability

The datasets generated during the current study are available from the corresponding author upon reasonable request.
