# Supplementary material for: Peptide-functionalized periodic mesoporous silica nanoparticles for monocyte-specific TET3 Silencing enhance cardiac repair after acute myocardial infarction
Source: J Nanobiotechnology. 2025 Nov 26;23:743. doi: 10.1186/s12951-025-03782-4 (PMC12659391; doi:10.1186/s12951-025-03782-4)
Supplement: Supplementary file 2 — Supplementary Material 2. [file 12951_2025_3782_MOESM2_ESM.docx]

**Peptide-functionalized periodic mesoporous silica nanoparticles for monocyte-specific TET3 silencing enhance cardiac repair after acute myocardial infarction**

Hao Jin^1,2,#^, Jiandong Ding^1,#^, Xiaoguo Zhang^1^, Shouquan Cheng^2^, Yahao Zhang^1^, Yong Wu^1^, Cihui Liu^3^, Sirui Yang^1^, Anjian Zhang^1^ and Genshan Ma^1^, Wenbin Lu^1,*^

^1^ Department of Cardiology, Zhongda Hospital Affiliated with Southeast University, Nanjing 210009, China;

^2^. Department of Cardiology, The Affiliated Hospital of Xuzhou Medical University, Xvzhou 221006, China;

^3^ Department of Biomedical Sciences, Nanjing Normal University, Nanjing 210023, China;

^*^ Correspondence: 101012092@seu.edu.cn;

^#^ These authors contributed equally to this work.

**1. Supplementary materials**

PMS was provided by the State Key Laboratory of Bioelectronics, School of Biological Science and Medical Engineering, Southeast University, China. CGTArg (Cys-Gly-Trp-Arg-Arg-Arg-NH_2_) was produced by Sangon Biotech (Shanghai, China) (Mass spectrometry analysis was shown in Figure S1A). Absolute ethanol, ultrapure water, methanol, phosphate buffer saline (PBS) were obtained from the Institute of Cardiovascular Diseases, Southeast University. Sigma-Aldrich (Germany) supplied the polyethylenimin (PEI, MW=25000 Da), guanidine hydrochloride (Guan-HCl, MW=95.53) and O-[N-(3-Maleimidopropionyl)-aminoethyl]-O-[3-(N-succinimi dyloxy)-3-oxopropyl]heptacosaethylene glycol (NHS-PEG-Mal). Solarboi Life Sciences (Beijing, China) provided TritonX-100 and lysosome-tracker red. We obtained non-fat powdered milk, BCA reagent, PageRuler Prestained Protein Ladder, 4% paraformaldehyde, Phenylmethanesulfonyl fluoride (PMSF), RIPA lysis buffer, ECL Plus Detection Kit, SDS-PAGE protein loading buffer and chemokine CCL2 from Biosharp (Hefei, China). We obtained cell culture plates from Coring (USA) in sizes 6, 12, 24, and 96 wells. ACE (Changzhou, China) provided the running buffer, transfer buffer and Servicebio (Wuhan China) provided tris buffered saline tween (TBST). Anti-CD14 for blocking CD14 was obtained from Hycult Biotech (Uden, The Netherlands). THP-1 (CL-0233) and HUVECs (CL-0675) were purchased from Procell (Wuhan, China). Collagenase I was obtained from Sigma (Missouri, USA) and deoxyribonuclease I was obtained from Roche (Basel, Switzerland). The siRNA sequences synthesized by GenePharma (Shanghai, China) for use in our research were as follows ^[1-3]^ : For humans：F: GGACAAUCCCAAAGAGGAATT, R: UUCCUCUUUGGGAUUGUCCTT; For mice：F:AGGCCAAGCUCUACGGGAATT, R:UUCCCGUAGAGCUUGGCCUTT; For pigs：F: CAGAAUGCCGUGAUUGUCAUCCUCA, R: UGAGGAUGACAAUCACGGCAUUCUG. In our research, the primary antibodies used for Western Blotting (WB) and immunofluorescence were obtained, including: IL-1β Proteintech (26048-1-AP), IL-6 Proteintech (26404-1-AP), CD31 Proteintech (11265-1-AP), CD31 Proteintech (28083-1-AP), VEGFa Proteintech (19003-1-AP), TET3 CST (99980S), TET3 abcam (ab153724). In our research, the main antibodies used for mouse flow cytometry were obtained from Biolegend (CA, USA), including FITC anti-mouse CD45 (157608), APC anti-mouse/human CD11b Antibody (101212), PE anti-mouse IL-6 Antibody (504504), PE anti-mouse Ly-6C Antibody (128008). The primary antibodies used for human flow cytometry in our research were obtained from Biolegend (CA, USA), including: FITC anti-human CD14 Antibody (325604) and PE anti-human CD16 (980102). The main antibodies used for pig flow cytometry in our research were obtained from BIO-RAD (CA, USA), including: FITC anti-pig CD14 (MCA1218F) and PE anti-pig CD163 (MCA2311PE). The specific primers used for the PCR experiments are listed in Table S1.

**2. Supplementary methods**

**2.1 Research on human**

**2.1.1 Analyzing the TET3 expression in AMI patients based on the GEO database.**

**(1) Acquisition and download of the GEO database**

The dataset GSE62646^[4]^ was obtained from the Gene Expression Omnibus (GEO), which included peripheral blood mononuclear cell samples from 28 ST elevation myocardial infarction (STEMI) patients and 14 stable coronary artery disease (SCAD) patients. The dataset was based on the GPL6244 platform, utilizing the [HuGene-1_0-st] subtype Affymetrix Human Gene 1.0ST Array [transcript (gene) version]. Gene expression matrices and platform data could be viewed and downloaded from the NCBI GEO database (https://www.ncbi.nlm.nih.gov/geo/). This study primarily focused on the inflammatory gene changes occurring within 24 hours of AMI onset, analyzing data from STEMI and SCAD patients upon admission. Moreover, the samples in this study consist of peripheral blood mononuclear cells. Based on the aforementioned dataset, our study primarily investigated changes in the TET family, especially TET3, in AMI patients.

**(2) Data processing and visualization analysis of differential genes**

The limma package in R (version 4.2.3) was utilized for data analysis, with |logFoldChange|≥ 0.4 and adjusted p < 0.05 considered significant differences. Differential expression genes were screened, with logFoldChange > 0.4 indicating upregulation and logFoldChange < 0.4 indicating downregulation among the differential genes. Differential genes were visualized using the ggplot2, ggpubr, and pheatmap packages in R. The ggplot2 package was employed to construct volcano plots of differential genes, the ggpubr package was used for constructing boxplots of differential genes, and the pheatmap package was utilized for constructing heatmaps of differential genes. Additionally, based on the obtained dataset, the R package ggpubr was used to further display the expression differences of TET3 in AMI patients at different time periods through violin plots. Subsequently, the R package pROC was employed to construct ROC curves, further evaluating the diagnostic value of TET3 for AMI risk.

**2.1.2 Analysis of the association between TET3 expression and AMI risk based on clinical data**

**(1) Study Population**

Patients who visited the Department of Cardiology at Zhongda Hospital, affiliated with Southeast University, between January 2022 and December 2022 were selected for this study. AMI patients who met the inclusion criteria were included as the study group, while SCAD (stable coronary artery disease) patients served as the control group. This study had been approved by the hospital's ethics committee (Approval No.: 2021ZDSYLL373-P01).

**(2) Inclusion Criteria and Exclusion Criteria**

The diagnosis of AMI was based on the Fourth Universal Definition of Myocardial Infarction. The diagnostic criteria for AMI required the presence of acute myocardial injury with clinical evidence of acute coronary syndrome, characterized by a rise and/or fall in cardiac troponin (cTn) levels, with at least one value exceeding the 99th percentile upper reference limit, along with at least one of the following: Symptoms of coronary artery disease; New ischemic electrocardiographic changes; Development of pathological Q waves; Imaging evidence of new loss of viable myocardium or new regional wall motion abnormality consistent with an ischemic etiology; Identification of coronary thrombosis via angiography.

Patients were excluded if they had any of the following conditions: atrial fibrillation; aortic dissection; pulmonary embolism; congenital heart disease; allergic asthma; major trauma or coexisting anemia; connective tissue disease; malignancy or hematologic disorders; immunosuppressants; severe infectious diseases.

**(3) Baseline Characteristics**

Baseline clinical data of the enrolled patients were retrieved from the hospital information system, including: General clinical information: gender, age, smoking history, and family history; Comorbidities: hypertension, hyperlipidemia, diabetes mellitus, and chronic kidney disease; Blood tests: Hb, BUN, Cr, UA, TG, and TCHO; Echocardiographic examination: LVEF; Medication: statins, antiplatelet agents, ACEI, β-blockers, and calcium channel blockers.

**(4) Detection of TET3 mRNA Expression in monocytes based on RT-PCR analysis**

Peripheral blood (5 mL) was collected from AMI patients (within 24 hours) and SCAD patients. Then, monocytes were obtained from the blood. Monocytes were lysed using TRIzol reagent, and RT-PCR was performed to detect TET3 mRNA expression.

**2.1.3 Association between TET3 expression and AMI prognosis based on clinical data**

**(1) Study Population**

AMI patients who underwent emergency treatment at Zhongda Hospital, affiliated with Southeast University, were enrolled in the study based on the inclusion criteria. All patients received either percutaneous coronary intervention (PCI) or conservative treatment. This study was approved by the hospital's ethics committee (Approval No.: 2021ZDSYLL373-P01).

**(2) Inclusion Criteria and Exclusion Criteria**

As previously described, the diagnosis of AMI was based on the Fourth Universal Definition of Myocardial Infarction, which included criteria such as typical chest pain symptoms, electrocardiographic changes, elevated serum troponin levels, and coronary angiographic findings; All AMI patients had been hospitalized for treatment.

Patients were excluded if they had any of the following conditions: atrial fibrillation; aortic dissection; pulmonary embolism; congenital heart disease; allergic asthma; major trauma with concurrent anemia; connective tissue disease; malignancy or hematologic disorders; immunosuppressants; severe infectious diseases.

**(3) Baseline Characteristics**

Baseline clinical data of the enrolled patients were retrieved from the hospital information system, including: General clinical information: gender, age, smoking history, and family history; Comorbidities: hypertension, hyperlipidemia, diabetes mellitus, and chronic kidney disease; Blood tests: Hb, BUN, Cr, UA, TG, and TCHO; Echocardiographic examination: LVEF; Medication: statins, antiplatelet agents, ACEI, β-blockers, and calcium channel blockers.

**(4) Coronary Intervention Data Collection and Gensini Score**

All patients underwent coronary angiography, which was performed by experienced interventional cardiologists. Imaging was acquired from multiple angles, ensuring that the left anterior descending (LAD) and left circumflex (LCX) arteries were visualized in at least four projections, while the right coronary artery (RCA) was visualized in at least two projections. Based on the imaging data, interventional parameters were extracted, including the culprit vessel, the site and severity of vascular stenosis, and intraoperative lesion characteristics. The obtained angiographic data were used to calculate the Gensini score, which was assessed independently by two experts in a blinded manner. In cases of discrepancy, a third expert was consulted to provide the final score. The Gensini score was calculated by multiplying the severity score of coronary artery stenosis by the location weighting factor. A higher Gensini score indicated more severe coronary artery disease. The Gensini scoring system assessed both the degree of stenosis and the location of the lesion as follows:

Severity scoring:1%–25% stenosis = 1 point; 26%–50% stenosis = 2 points; 51%–75% stenosis = 4 points;76%–90% stenosis = 8 points; 91%–99% stenosis = 16 points; Complete occlusion = 32 points; Location weighting factors:Left main coronary artery = 5.0; Proximal left anterior descending artery = 2.5; Mid left anterior descending artery = 1.5; Distal left anterior descending artery = 1.0; Proximal left circumflex artery = 2.5; Mid or distal left circumflex artery = 1.0; Right coronary artery = 1.0;Small branches = 0.5.

**(5) RT-PCR analysis of TET3 mRNA expression in monocytes**

5 mL of peripheral blood was collected from AMI patients within 24 hours of admission, and monocytes were isolated. The cells were lysed using TRIzol reagent, and RT-PCR was performed to detect TET3 mRNA expression.

**(6) Follow-Up and Endpoint Event Recording**

This study focused on in-hospital risk assessment, meaning that follow-up began at hospital admission and ended at discharge. Cardiovascular events that occurred during hospitalization were recorded, with a maximum follow-up period of two weeks. Follow-up was conducted daily, and data collection ceased after the two-week period. Only endpoint events were recorded, without further post-discharge follow-up. The primary endpoint events in AMI patients included the occurrence of major adverse cardiovascular events (MACE), which were defined as follows: all-cause mortality, cardiogenic shock, cardiac arrest, recurrent myocardial infarction, in-stent thrombosis, repeat revascularization, malignant arrhythmias (including ventricular flutter, ventricular fibrillation, and high-degree atrioventricular block, acute stroke.

**2.2 Synthesis and Characterization of PMS-siTET3-PEI-PEG/PEI-PEG-CGTArg**

**2.2.1 Preparation of PMS-siTET3-PEI-PEG/PEI-PEG-CGTArg**

The delivery system was constructed referred to the previous design ^[5-7]^.

0.5 mg of PMS, 80 μL of anhydrous ethanol and 20 μL of 4M Guanidine Hydrochloride (Guan-HCl) solution were added to a 1.5 mL centrifuge tube. The mixture was then dispersed using ultrasound (80 W). Following this, 10 μL of 0.1 nmol/μL siTET3 solution was added, and dispersion was carried out with ultrasound for 15 minutes. Subsequently, the mixture was shaken at 4°C for 1h. Afterward, centrifugation was performed at 4°C and 12000 rpm for 10 minutes to obtain precipitated particles (PMS-siTET3).

An ethanol solution of PEI/PEI-PEG was prepared at a concentration of 1 mg/mL. The precipitated particles (PMS-siTET3) obtained from the previous step were washed with ethanol, then resuspended in 200 μL of anhydrous ethanol and dispersed using ultrasound. Dropwise addition of an equal volume of 1 mg/mL PEI/PEI-PEG ethanol solution was carried out, which was dispersed with ultrasound for 15 minutes. After centrifugation and removal of the supernatant, PMS-siTET3-PEI/PEI-PEG particles were obtained, which were washed with water (pH=5.0). The particles were then washed and suspended in anhydrous ethanol.

Solutions were prepared containing NHS-PEG-Mal at a concentration of 8 mg/mL in DMSO, and CGTArg solution at a concentration of 1 mg/mL in water. 50 μL of 8 mg/mL NHS-PEG-Mal solution was mixed with 20 μL of 1 mg/mL CGTArg solution, which was vortexed and then left at 37°C for 30 minutes to form NHS-PEG-CGTArg. NHS-PEG-CGTArg was then added to 100 μL of PMS-siTET3-PEI/PEI-PEG and vortexed at room temperature for 30 minutes. After centrifugation at 12000 rpm for 10 minutes and removal of the supernatant, the particles were washed with water. Finally, the particles were resuspended in 500 μL of water to obtain a solution of 1 µg/µL PMS-siTET3-PEI-PEG/PEI-PEG-CGTArg (**Figure 1A1** and **Figure** **1A2**).

**2.2.2 Evaluation the loading of siTET3 in the particles**

Four independent 1.5 mL centrifuge tubes were used for the experiment. In each tube, 5, 10, 15 and 20 μL of 0.1 nmol/μL siTET3 aqueous solution were added, which was added to 20µL respectively. To each tube, 80 μL of water and 20 μL of 4 M Guan-HCl solution were added. The resulting mixture was subjected to ultrasonic dispersion and shaken at 4°C for 1h. Subsequently, the mixture was centrifuged, yielding precipitated particles and a supernatant solution. The concentration of siTET3 in the supernatant was measured using the NanoDrop 2000 (Thermo, The USA).

**2.2.3 Evaluation of the system stability**

To evaluate the protection of siTET3 by the delivery system, the different experiments were conducted.

Group 1: siTET3 was dispersed in water; Group 2: siTET3 was dispersed in water and incubated with ribonuclease (RNase) for 30 minutes; Group 3: PMS-siTET3-PEI-PEG/PEI-PEG-CGTArg was dispersed in water; Group 4: PMS-siTET3-PEI-PEG/PEI-PEG-CGTArg was dispersed in water and incubated with RNase for 30 minutes; Group 5: PMS-siTET3-PEI-PEG/PEI-PEG-CGTArg was treated with RNase, then incubated with heparin for 30 minutes; Group 6: PMS-siTET3-PEI-PEG/PEI-PEG-CGTArg was treated RNase, then co-incubated with heparin and RNase for 30 minutes. After incubation, all mixtures were centrifuged to collect the supernatant. The collected supernatants were loaded into a 1% agarose gel with 0.01% GoldView staining and run with TAE running buffer at 140 V for 40 minutes. The gel was then visualized using a UV illuminator (254 nm) and images were captured using a Bio-Rad imaging system (Hercules, CA).

To further simulate the release of nanomaterials in the lysosomal environment, a PBS buffer at pH = 5.0 was prepared using disodium hydrogen phosphate. To mimic the biochemical conditions of intracellular lysosomes, 1 mg of dihydrogen phosphate (a type of phosphatase) was added per 100 mL of the pH = 5.0 buffer solution. Meanwhile, a PBS buffer at pH = 7.4 was prepared using disodium hydrogen phosphate to simulate the physiological fluid circulation environment. Subsequently, 1 mg of PMS-siTET3-PEI-PEG/PEI-PEG-CGTArg was dispersed in each of the above solutions, and the release amount of siTET3 was measured at 12 h, 24 h, and 48 h. The concentration of siTET3 in the supernatant was measured using the NanoDrop 2000 (Thermo, USA).

**2.2.4. Characterization of the particles**

The particles, including PMS, PMS-siTET3-PEI/PEI-PEG and PMS-siTET3-PEI-PEG/PEI-PEG-CGTArg, dispersed in water, were subjected to morphological and structural analysis using a transmission electron microscope (TEM) (JEM-2100; JEOL, Tokyo, Japan). The zeta potential of the particles was measured using a ZetaSizer Nano instrument (Malvern Instruments Ltd., Worcestershire, UK).

The determination of CGTArg was conducted by forming a complex between peptide bonds and Cu^+^ in an alkaline solution. This complex reacts with the reagent in the BCA assay to produce a colored compound at 562nm wavelength. The amount of this compound generated was directly proportional to the protein concentration. Based on this principle, a standard curve of CGTArg concentration versus absorbance was established using the BCA assay kit. Subsequently, the CGTArg content on particles was determined relying on the standard curve and the BCA reagent.

The surface of the particles was coated with gold, and the particles were characterized using SEM (TESCAN, Brno, Czech Republic) at an acceleration voltage of 20 kV. Additionally, energy-dispersive spectrometry (EDS) was employed for further characterization of the particles.

**2.3. Research on cells**

**2.3.1. Cell culture**

We utilized THP-1 cells as monocytes in our studies. THP-1 cells were cultured using 1640 cell culture medium. HUVECs were cultured using DMEM cell culture medium. All cell cultures were maintained in a cell incubator at 37°C with 5% CO2.

**2.3.2. Evaluation of biocompatibility** **and transfection efficiency**

**(1) Toxicity of the particles to monocytes**

100 μL of THP-1 monocytes at a concentration of 10^6^ cells/mL were seeded into each well of a 96-well plate. Different particles, including PMS, PMS-siTET3-PEI-PEG/PEI and PMS-siTET3-PEI-PEG/PEI-PEG-CGTArg (0, 20, 40, 60, 80, and 100 μg/mL), were added to the respective wells. The cells in wells not treated with particles served as the control group. Cells were treated with 2% Triton X-100 as the positive control group. The plates were then placed in a suspension cell culture incubator.

Cell viability was assessed at 24 and 72h after co-incubation by using the CCK-8 reagent. The samples were measured using an automatic microplate reader (Thermo Fisher, MA, USA) at a wavelength of 450 nm. Cell viability was calculated using the following formula

$$Cell viability(\%)=\frac{\mathrm{OD}\mathrm{test}-OD\mathrm{blank}}{\mathrm{OD}\mathrm{control}-OD\mathrm{blank}}*100\%$$

**(2) Red blood cell biocompatibility of the particles**

A total of 100 µL of blood was collected from AMI mice. The blood sample was then centrifuged at 1000 rpm for 5 minutes, and the supernatant was carefully removed. RBCs were washed five times with normal saline (NS), and 4 mL of NS was added to achieve a red cell suspension (RCS).

Next, 900 µL of RCS was transferred to 1.5 mL centrifuge tubes. To the mixture, 100 µL of NS (negative control), water (positive control group), PMS- siTET3- PEI-PEG/PEI (prepared with NS) and PMS-siTET3-PEI-PEG/PEI-PEG-CGTArg (prepared with NS) were added. The mixture was then incubated for 2h at 37°C. After incubation, the mixture was centrifuged at 1000 rpm for 10 minutes, and 100 µL of supernatant was collected for absorbance measurement at 570 nm.

$$\mathrm{Hemolysis}\left（ \% \right）=\frac{\mathrm{OD}\mathrm{test}-OD\mathrm{negative}}{\mathrm{OD}\mathrm{positive}-OD\mathrm{negative}}*100\%$$

Furthermore, the sediment obtained after centrifugation was collected and observed using SEM to examine the morphological characteristics of RBCs.

**(3) Transfection efficiency of the particles**

THP-1 monocytes were plated at a density of 10^6^ cells per well. Fresh medium was replaced when the cell density reached 80%. Subsequently, PMS-fam-siTET3-PEI-PEG/PEI-PEG-CGTArg at concentrations of 10 µg/ml and 20 µg/ml were added, which was incubated with the cells for 72h in a suspension cell incubator. After the cells were washed and dispersed with PBS, transfection efficiency was determined using flow cytometry (BD, USA).

**2.3.3. Targeting ability and lysosomal escape of PMS-siTET3-PEI-PEG/PEI-PEG-CGTArg**

THP-1 monocytes were seeded. Subsequently, 20 µg/ml PMS-fam-siTET3-PEI-PEG/PEI-PEG-CGTArg (labeled with fam) was added and co-incubated with cells for 6h, 24h and 72h in the suspension cell incubator. Afterwards, the original medium was discarded, and the cell mixture was washed with PBS. Hoechst dye was added for 30 minutes, followed by three washes with PBS. Fluorescence images were captured using confocal laser scanning microscopy (CLSM) with a Zeiss LSM710 microscope (Olympus, Japan).

In comparison, after treated with lipopolysaccharide (LPS) to simulate the inflammation in MI, monocytes were incubated with PMS-fam-siTET3-PEI-PEG/PEI-PEG-CGTArg for 6h, 24h, 72h and 72h (monocytes were treated with anti-CD14 (3ug/ml) for blocking CD14 for 30 min before co-incubation with PMS-fam-siTET3-PEI-PEG/PEI-PEG-CGTArg) in the suspension cell incubator. In the subsequent steps, the original medium was discarded, and medium containing lysosomal fluorescent probes (Red) was added and incubated for 2h. Then monocytes were stained with Hoechst and observed with CLSM.

**2.3.4. Chemotaxis of monocytes treated with the delivery system**

Using a 12-well transwell chamber (Millipore), we examined whether the delivery system affected the chemotaxis of monocytes. HUVECs were seeded in the upper chamber. Group 1: Monocytes were co-incubated with PBS for 24 hours, seeded in the upper chamber, and medium with PBS was added to the lower chamber as the control group; Group 2: Monocytes were co-incubated with PBS for 24 hours, seeded in the upper chamber, and medium with CCL2 was added to the lower chamber; Group 3: Monocytes were co-incubated with PMS-siTET3-PEI-PEG/PEI-PEG-CGTArg for 24 hours, seeded in the upper chamber, and medium with PBS was added to the lower chamber; Group 4: Monocytes were co-incubated with PMS-siTET3-PEI-PEG/PEI-PEG-CGTArg for 24 hours, seeded in the upper chamber, and medium with CCL2 was added to the lower chamber. After seeding, the transwell systems were placed in cell culture incubators for 12 hours.

Subsequently, CCK-8 reagent was added to each well, and the transwell system was incubated at 37°C for 40 minutes. The mixture was transferred to a 96-well plate and measured using a microplate spectrophotometer at 450 nm. To compare the chemotaxis effects of monocytes in different groups, we used the blank group as a reference value of 0, and the control group as a reference value of 1. Additionally, the monocytes transferred to the lower chamber were stained with crystal violet for observation.

$$Chemotactic effect=\frac{\mathrm{OD}\mathrm{test}-OD\mathrm{blank}}{\mathrm{OD}\mathrm{control}-OD\mathrm{blank}}$$

**2.3.5. Western blot and Flow cytometric analysis**

After stimulated with LPS, monocytes were plated at a density of 10^6^ cells per well. Subsequently, PBS, PMS-siNC-PEI-PEG/PEI-PEG-CGTArg (20 µg/ml) and PMS-siTET3-PEI-PEG/PEI-PEG-CGTArg (20 µg/ml) were added and co-incubated with monocytes for 72h. In comparison, PMS-siTET3-PEI-PEG/PEI-PEG-CGTArg (20 µg/ml) was also added and co-incubated with monocytes for 48h. Monocytes not stimulated with LPS were served as the control group. After treatment, WB analysis was performed using an anti-TET3 antibody (CST, USA) and corresponding secondary antibodies (Proteintech, China)

Similar to the previous co-incubation, the monocytes were washed three times with cold PBS and resuspended at a concentration of 10^7^ cells/mL. A 100 µL of the cell suspension was prepared. Afterwards, the cell suspension were incubated with FITC-anti-human CD14 (Biolegend, CA, USA) and PE-anti-human CD16 antibodies (Biolegend, CA, USA) for 30 minutes at 4°C. Subsequently, different subtypes of monocytes were identified using a FACS Calibur flow cytometer (BD, USA).

**2.4.** **Research on mice**

**2.4.1. Animals**

Male ICR mice, aged 7–8 weeks, were obtained from Cavens Co., Ltd. in Nanjing, China, and were housed in a specific pathogen-free facility. The mice were maintained in an environment with a 12-hour light/12-hour dark cycle and a constant temperature of 22℃. The animal study protocol underwent thorough review and received approval from the Southeast University Animal Welfare Committee (No: 20240226003).

**2.4.2. Animal Model**

The AMI model was induced by ligating the left anterior descending coronary artery, and confirmation was obtained through ST-segment-characterized electrocardiogram analysis and observation of color alteration in the left ventricle. Then, AMI mice were then randomly assigned to different groups, which would be treated with different particles in the following experiment. For the drug administration protocol, the nanoparticles (25 μg/g) were administered via tail vein injection immediately upon the occurrence of AMI.

**2.4.3. Pharmacokinetic, biodistribution and targeting specificity of PMS-siTET3-PEI-PEG/PEI-PEG-CGTArg**

**(1) Pharmacokinetic study of PMS-siTET3-PEI-PEG/PEI-PEG-CGTArg**

In a pharmacokinetic study, healthy mice (N= 3) were intravenously administered PMS-siTET3 (labeled with Cy5)-PEI-PEG/PEI-PEG-CGTArg via the tail vein (25 μg/g). Blood samples (30 µL) were collected at various intervals (1 min, 1 h, 2 h, 3 h, 4 h, 12 h, 24h and 48 h post-injection) via cheek pouch puncture to assess circulating profiles. Fluorescent imaging of the blood samples was performed using an in vivo imaging system (IVIS) imaging system (PerkinElmer, Inc., Waltham, MA, USA) with excitation at 650 nm and emission at 670 nm. Data analysis was conducted using Living Image software (Version 4.4). Data at each time point were normalized relative to the initial time point (1 min post-injection) to account for individual variability.

**(2) Biodistribution of PMS-siTET3-PEI-PEG/PEI-PEG-CGTArg**

For the biodistribution study, healthy mice were divided into two groups (N= 3) and intravenously administered PMS-siTET3 (labeled with Cy5)-PEI-PEG/PEI-PEG-CGTArg via the tail vein for 12h and 72h, respectively. After the treatment, mice were anesthetized and sacrificed, and major organs (liver, spleen, lung, kidney, brain and heart) were harvested for ex vivo imaging using IVIS. Data processing was performed with Living Image software.

**(3) Targeting MI area of PMS-siTET3-PEI-PEG/PEI-PEG-CGTArg**

To assess the targeting specificity of PMS-siTET3-PEI-PEG/PEI-PEG-CGTArg in the MI area, AMI mice were divided into two groups (N=3) and subjected to different treatment regimens. The first group received PMS-siTET3 (labeled with Cy5)-PEI-PEG/PEI-PEG-CGTArg treatment for 12h and the second group received PMS-siTET3 (labeled with Cy5)-PEI-PEG/PEI-PEG-CGTArg treatment for 72h. After the treatment, mice were euthanized under anesthesia, PBS perfusion was performed, and cardiac tissues were collected and examined using IVIS. Subsequent data analysis was conducted using Living Image software.

**(4) Targeting monocytes of PMS-siTET3-PEI-PEG/PEI-PEG-CGTArg in vivo**

AMI mice were intravenously injected with fluorescently labeled nanoparticles PMS(labeled with DiR)-siTET3-PEI-PEG/PEI-PEG-CGTArg via the tail vein. Blood was collected 6 hours post-injection to assess the uptake of nanoparticles by different cells. The collected blood was lysed using red blood cell lysis buffer. After complete lysis, the cell suspension was filtered through a 70 μm strainer to purify the cells. The cells were then resuspended and subjected to flow cytometry analysis. The antibodies used included: Brilliant Violet 605™ anti-mouse CD45, APC anti-mouse CD11b, FITC anti-mouse CD115, and PE anti-mouse Ly6G. All flow cytometry antibodies were purchased from BioLegend.

Furthermore, to further assess the targeting specificity of PMS-siTET3 (labeled with fam)-PEI-PEG/PEI-PEG-CGTArg on monocytes in vivo, whole mouse blood was obtained from AMI mice of three groups. The blood samples were co-incubated with particles for different time: PMS-siTET3-fam-PEI-PEG/PEI-PEG-CGTArg (30 min), PMS-siTET3-fam-PEI-PEG/PEI-PEG-CGTArg (1 h), PMS-siTET3-fam-PEI-PEG/PEI-PEG-CGTArg (1 h) (whole blood was blocked with anti-CD14 for 30min before co-incubation with PMS-siTET3-fam-PEI-PEG/PEI-PEG-CGTArg). After the treatment, monocytes were extracted from the blood and subjected to nuclear staining with Hoechst, which was then observed using a fluorescence microscope. Data analysis was conducted using ImageJ software.

**2.4.4. Expression of TET3 in AMI**

AMI mice and healthy mice (N=3) were fully anesthetized and euthanized. Subsequently, blood was collected and monocytes were isolated. Following this, monocytes were obtained for WB and immunofluorescence analysis to assess the expression levels of TET3. TET3 (CST, USA) was used for WB analysis and TET3 (Abcam, UK) was used for immunofluorescence analysis. And corresponding secondary antibodies were used.

**2.4.5. PMS-siTET3-PEI- PEG/PEI-PEG-CGTArg reprograming monocytes**

AMI mice were randomly divided into four groups (N=3) and received tail vein injections of PBS, PMS-PEI-PEG/PEI-PEG-CGTArg, PMS-siNC-PEI-PEG/PEI-PEG-CGTArg and PMS-siTET3-PEI-PEG/PEI-PEG-CGTArg, respectively. After 72h, mice were euthanized, and 1ml of blood was collected.

Mononuclear cells were obtained from the blood. Flow cytometry analysis was conducted on mononuclear cells to evaluate the impact of PMS-siTET3-PEI-PEG/PEI-PEG-CGTArg on the proportions of inflammatory and anti-inflammatory monocytes. Specific flow cytometry antibodies including APC-anti-CD11b (Biolegend, USA), PE-anti-Ly6C (Biolegend, USA), PE-anti-IL-1β (Biolegend, USA), and PE-anti-IL-6 (Biolegend, USA) were utilized for cell staining. Following staining, cells were analyzed using Flow cytometry (BD, USA).

In addition, monocytes were obtained from the blood and subjected to immunofluorescence using antibodies, including anti-TET3 (Proteintech, China), anti-Ly6C (Proteintech, China), anti-IL-1β (Proteintech, China), and anti-IL-6 (Proteintech, China).

Similarly, monocytes were obtained for WB analysis to evaluate the effect of PMS-siTET3-PEI-PEG/PEI-PEG-CGTArg on inflammatory proteins. The impact of PMS-siTET3-PEI-PEG/PEI-PEG-CGTArg on inflammatory markers was evaluated using specific antibodies, including anti-TET3 (Proteintech, China), anti-Ly6C (Proteintech, China), anti-IL-1β (Proteintech, China) and anti-IL-6 (Proteintech, China).

**2.4.6 Effect of PMS-siTET3-PEI- PEG/PEI-PEG-CGTArg on chemotaxis of monocytes**

AMI mice were randomly divided into four groups (N=3) and received tail vein injections of PBS, PMS-PEI-PEG/PEI-PEG-CGTArg, PMS-siNC-PEI-PEG/PEI-PEG-CGTArg and PMS-siTET3-PEI-PEG/PEI-PEG-CGTArg, respectively. After 72h, mice were euthanized, and 1ml of blood was collected. Similarly, monocytes were obtained. Then the expression of C-C chemokine receptor (CCR2) in monocytes was analyzed with WB and immunofluorescence. The primary antibodies used were anti-CCR2 (Proteintech, China).

**2.4.7 Effect of PMS-siTET3-PEI- PEG/PEI-PEG-CGTArg on the distribution of monocytes/macrophages**

AMI mice were randomly divided into four groups (N=3) and received tail vein injections of PBS, PMS-PEI-PEG/PEI-PEG-CGTArg, PMS-siNC-PEI-PEG/PEI-PEG-CGTArg and PMS-siTET3-PEI-PEG/PEI-PEG-CGTArg. After a three-day treatment period, the phenotypes of cardiac macrophages were assessed using immunofluorescence.

In brief, cardiac tissues were extracted, embedded, and sectioned for immunofluorescence staining. The tissue sections, embedded in paraffin, were sliced near ligatures. After deparaffinization and gradient alcohol dehydration, antigen retrieval was performed, followed by PBST washing, blocking with 2% BSA, and overnight incubation with specific primary antibodies. Subsequent steps included PBST washing, incubation with secondary antibodies, further PBST washing, and fluorescence observation. The specific antibodies utilized included anti-CD11b (Proteintech, China) anti-cTnT (Proteintech, China).

**2.4.8. Effect of PMS-siTET3-PEI- PEG/PEI-PEG-CGTArg on inflammatory cytokines in serum**

Similarly, AMI mice were treated with PBS, PMS-PEI-PEG/PEI-PEG-CGTArg, PMS-siNC-PEI-PEG/PEI-PEG-CGTArg and PMS-siTET3-PEI-PEG/PEI-PEG-CGTArg (N=3). Three days after the treatment, a 1ml blood sample was collected from the AMI mice and centrifuged at 3000rpm for 30 minutes to separate the plasma. The obtained plasma was carefully stored in a -80°C freezer to maintain its integrity. To assess the concentrations of inflammatory cytokines IL-1β and IL-6 in the serum, ELISA kits were utilized (mlbio, China).

**2.4.9. Cardiac Protection of PMS-siTET3-PEI-PEG/PEI-PEG-CGTArg**

**(1) Impact on infract area of PMS-siTET3-PEI-PEG/PEI-PEG-CGTArg**

AMI mice were randomly divided into four groups (N=3) and received tail vein injections of PBS, PMS-PEI-PEG/PEI-PEG-CGTArg, PMS-siNC-PEI-PEG/PEI-PEG-CGTArg and PMS-siTET3-PEI-PEG/PEI-PEG-CGTArg, respectively. 72h post-treatment, mice were deeply anesthetized and euthanized. The hearts were perfused with PBS, then harvested for 2,3,5-triphenyltetrazolium chloride (TTC) staining to assess the impact of PMS-siTET3-PEI-PEG/PEI-PEG-CGTArg on MI area. In brief, mouse hearts were rapidly frozen at -20°C for 10 minutes, followed by 2mm sectioning. The obtained heart slices were stained with 1% TTC for 10-30 minutes and photographed to observe the MI area.

**(2) Impact on LVEF of PMS-siTET3-PEI-PEG/PEI-PEG-CGTArg**

Similarly, AMI mice were treated with PBS, PMS-PEI-PEG/PEI-PEG-CGTArg, PMS-siNC-PEI-PEG/PEI-PEG-CGTArg and PMS-siTET3-PEI-PEG/PEI-PEG-CGTArg, respectively (N=3). Then, AMI mice underwent echocardiography at both 3 days and 30 days to evaluate the influence of PMS-siTET3-PEI-PEG/PEI-PEG-CGTArg on left ventricular ejection fraction (LVEF).

**(3) Impact on myocardial fibrosis of PMS-siTET3-PEI-PEG/PEI-PEG-CGTArg**

AMI mice were treated with PBS, PMS-PEI-PEG/PEI-PEG-CGTArg, PMS-siNC-PEI-PEG/PEI-PEG-CGTArg and PMS-siTET3-PEI-PEG/PEI-PEG-CGTArg, respectively (N=3). After one month of treatment, mice were anesthetized and euthanized. Following PBS perfusion, mouse heart tissues were collected and fixed in paraformaldehyde for assessment of myocardial fibrosis. Briefly, mouse hearts were embedded in paraffin, sliced near the ligature, and subjected to Masson's trichrome staining. In short, tissue sections were dehydrated using xylene and ethanol, stained with hematoxylin for 10 minutes, rinsed, and differentiated with hydrochloric acid. Masson's trichrome staining was then performed for 10 minutes.

**(4) Impact on border zone cardiomyocytes of PMS-siTET3-PEI-PEG/PEI-PEG-CGTArg**

AMI mice were treated with PBS, PMS-PEI-PEG/PEI-PEG-CGTArg, PMS-siNC-PEI-PEG/PEI-PEG-CGTArg and PMS-siTET3-PEI-PEG/PEI-PEG-CGTArg, respectively (N=3). After one month of treatment, mouse heart tissues were collected for hematoxylin-eosin (HE) staining.

**(5) Impact on angiogenesis of PMS-siTET3-PEI-PEG/PEI-PEG-CGTArg**

Similarly, AMI mice were treated with PBS, PMS-PEI-PEG/PEI-PEG-CGTArg, PMS-siNC-PEI-PEG/PEI-PEG-CGTArg, and PMS-siTET3-PEI-PEG/PEI-PEG-CGTArg. After one month, mouse cardiac tissue was collected to evaluate the impact of PMS-siTET3-PEI-PEG/PEI-PEG-CGTArg on CD31 and VEGFa protein levels. WB were performed with specific antibodies including, anti-CD31 (Proteintech, China) and VEGFa (Proteintech, China). In addition, heart sections were obtained for immunofluorescence analysis. The staining process was completed with CD31 and VEGFa immunofluorescence staining on heart sections, using the specific antibody anti-CD31 (Proteintech, China) and VEGFa (Proteintech, China).

**2.4.10. Validation of overexpression following nanoparticle therapy**

Seventy-two hours after the administration of PMS-siTET3-PEI-PEG/PEI-PEG-CGTArg in AMI mice, overexpression validation was repeated. The mice were randomly divided into three groups: one treated with PBS as the control group, and the others receiving PMS-PEI-PEG/PEI-PEG-CGTArg and PMS-PEI-PEG/PEI-PEG-CGTArg (pTET3). The sequence used for TET3 overexpression was based on previous studies [7].

The specific preparation methods followed established protocols[8-10]. Seventy-two hours post-treatment, peripheral blood was collected from the mice and used to evaluate monocyte subpopulations. Immunofluorescence was performed to detect TET3 expression, and flow cytometry was used to assess the levels of pro-inflammatory monocytes.

**2.4.11. Mechanism of tet3 in regulating the notch signaling pathway in monocytes**

Existing studies suggest that alterations in TET3 expression may be associated with the activation of the Notch pathway. To further investigate the underlying mechanism, we designed relevant experiments. AMI mice were injected via the tail vein with PMS-siNC-PEI-PEG/PEI-PEG-CGTArg and PMS-siTET3-PEI-PEG/PEI-PEG-CGTArg. After 48 hours, monocytes were isolated from the mice and sent to Majorbio (Shanghai, China) for transcriptomic analysis under strict low-temperature conditions. Differentially expressed genes were analyzed, with particular focus on key genes in the Notch pathway. Subsequently, we conducted PCR experiments to further validate these findings. Another group of AMI mice received tail vein injections of PBS, PMS-PEI-PEG/PEI-PEG-CGTArg, PMS-siNC-PEI-PEG/PEI-PEG-CGTArg, and PMS-siTET3-PEI-PEG/PEI-PEG-CGTArg. Monocytes were collected 48 hours later for PCR analysis to assess differential gene expression. Primer sequences used are provided in the supplementary materials.

**2.4.12 Off-Target Effect Detection of siTET3**

As described above, AMI mice were randomly divided into two groups (N=3) and injected via the tail vein with PMS-siNC-PEI-PEG/PEI-PEG-CGTArg and PMS-siTET3-PEI-PEG/PEI-PEG-CGTArg, respectively. At 72 hours post-treatment, the mice were euthanized, and 1 mL of blood was collected from each mouse. Monocytes were then isolated for transcriptome sequencing.

To evaluate the potential off-target effects of siTET3 treatment, bioinformatic analysis was performed using R software (version 4.2.3). First, differentially expressed genes (DEGs) between the siTET3 and siNC groups were identified using DESeq2 (threshold: FDR < 0.05 and |log2FC| > 1). Subsequently, the seed region (2–8 nt: GGCCAAG) of the siTET3 sequence and its reverse complement were extracted. Using the TxDb.Mmusculus.UCSC.mm10.knownGene and BSgenome.Mmusculus.UCSC.mm10 packages, all mouse gene 3′ UTR regions were scanned for transcripts containing this seed sequence or its complementary sequence. Genes corresponding to these transcripts were defined as potential off-target genes. Finally, the overlap between the DEGs and the potential off-target genes was identified, yielding a set of statistically significant off-target genes mediated by the siRNA seed region.

Subsequently, experimental validation was conducted. AMI mice were randomly divided into four groups (N=3) and injected via the tail vein with PBS, PMS-PEI-PEG/PEI-PEG-CGTArg, PMS-siNC-PEI-PEG/PEI-PEG-CGTArg, or PMS-siTET3-PEI-PEG/PEI-PEG-CGTArg. The newly designed siTET3 sequences used were: GCUCCAACGAGAAGCUAUUUUG; CGAGGUUGCUCUUCGAUAAAAC^[8]^. At 48 hours after treatment, the mice were euthanized, and 1 mL of blood was collected from each mouse. Monocytes were isolated for PCR analysis to evaluate differential gene expression.

**2.4.13. Safety Assessment of PMS-siTET3-PEI-PEG/PEI-PEG-CGTArg in mice**

Healthy mice (N=3) were intravenously injected with PMS-siTET3-PEI-PEG/PEI-PEG-CGTArg through the tail vein. Peripheral blood samples (30 µL) were collected from the mice before injection and at 0.5h, 2h, 24h, 72h and 1week post-injection to measure blood parameters, including aspartate aminotransferase (AST), alanine aminotransferase (ALT), creatinine (Cr), blood urea nitrogen (BUN), white blood cells (WBC), red blood cells (RBC), hemoglobin (HGB), platelets (PLT), IL-1β and IL-6.

In addition, our study assessed the major organ toxicity of PMS-siTET3-PEI-PEG/PEI-PEG-CGTArg at 1 week and 1 month post-injection. Mice were divided into four groups, receiving different treatments: PBS treatment for 1 month, PMS-siTET3-PEI-PEG/PEI-PEG-CGTArg treatment for 1 week, PMS-siTET3-PEI-PEG/PEI-PEG-CGTArg treatment for 1 month and repeated injections of PMS-siTET3-PEI-PEG/PEI-PEG-CGTArg for one month (Twice a month), PMS-siTET3-PEI-PEG/PEI-PEG-CGTArg treatment for 3 months; Subsequently, major organs including the liver, spleen, lung, kidney and brain were collected, processed into paraffin sections, and subjected to HE staining.

One month after administering the nanoparticles or PBS to AMI mice, serum was collected and preserved. Subsequently, nanoparticle antibody titers were evaluated via ELISA detection. The nanoparticles (1 mg) were distributed into 1.5 mL centrifuge tubes, followed by the addition of serum, while the control group received PBS. After specific binding of the primary antibody, the supernatant was removed by centrifugation. Finally, absorbance was measured using an ELISA secondary antibody.

**2.5. Research on Pigs**

**2.5.1. Animals**

5-7-month-old Bama Fragrant Pigs (20-25kg) were raised at 20-25°C with free access to feed. The pigs passed the quarantine inspection and were in good physical condition. The animal study protocol underwent thorough review and received approval from the Southeast University Animal Welfare Committee (No: 20240815001).

**2.5.2. AMI animal model**

The pig was placed in a supine position on the operating table. After local skin preparation, the area was disinfected with povidone-iodine and covered with sterile drapes. Local anesthesia was administered. The point with the strongest vascular pulsation was identified below the right groin, and femoral artery puncture was performed. After successful puncture, a guidewire was inserted, and a 6F arterial sheath was placed. A bolus of 5,000 units of heparin sodium was administered, followed by an additional 1,000 units per hour. Subsequently, under the guidance of an angiographic guidewire, a contrast catheter was advanced to the aortic root via the femoral artery. Ultravist-370 was injected to perform left and right coronary angiography, allowing clear analysis of the coronary artery distribution. The vessel was occluded using a balloon while avoiding the left anterior descending branch. A 6F JL (Judkins Left) guiding catheter was inserted into the coronary artery. After confirmation by angiography, the balloon was inflated using a pressure pump at 3–6 atm to perform ischemic preconditioning three times. Each balloon inflation lasted 20 seconds, with intervals of 3–5 minutes. No abnormal reactions were observed. The balloon was then expanded for one hour. Angiography showed interruption of blood flow distal to the balloon, and electrocardiographic monitoring indicated ST-segment elevation. After successful establishment of the AMI model, the balloon catheter and guidewire were withdrawn. The drug was slowly injected via a microcatheter. Upon completion, the balloon catheter and guidewire were removed. The arterial sheath was withdrawn, and the puncture site was promptly ligated and compressed for 20 minutes to achieve hemostasis. The wound was then sutured and the skin closed.

Upon successful establishment of the model, AMI pigs were then randomly assigned to different groups, and pigs were treated with different particles by intracoronary injection through the aortic sinus (1mg/kg).

**2.5.3. Pharmacokinetic evaluation of PMS-siTET3-PEI-PEG/PEI-PEG-CGTArg**

To further evaluate the metabolism of the delivery system in pigs, PMS-siTET3-PEI-PEG/PEI-PEG-CGTArg was injected via ear vein of pigs. Peripheral blood samples (200 μL) were collected at 0h, 0.5 h, 1 h, 2 h, and 24 h after injection. The silicon element of PMS-siTET3-PEI-PEG/PEI-PEG-CGTArg in the blood was measured using atomic absorption spectroscopy to further assess the metabolic characteristics of the delivery system in the body at different time intervals.

**2.5.4. PMS-siTET3-PEI-PEG/PEI-PEG-CGTArg reprograming monocytes**

After AMI pigs (N=4) treated with PMS-siTET3-PEI-PEG/PEI-PEG-CGTArg or PMS-siNC-PEI-PEG/PEI-PEG-CGTArg, peripheral blood samples were collected from the pigs. Subsequently, extraction of peripheral blood mononuclear cells (PBMCs) from the blood were obtained. Flow cytometry staining was performed to evaluate the proportions of different types of monocytes using CD14-FITC (BIO-RAD, USA) and CD163-PE (BIO-RAD, USA) antibodies.

Similarly, three days following PMS-siTET3-PEI-PEG/PEI-PEG-CGTArg (N=4) injection, a 5ml peripheral blood sample was collected from the pigs and centrifuged at 3000rpm for 30 minutes to separate the plasma. The obtained plasma was carefully stored in a -80°C freezer to maintain its integrity. To assess the concentrations of inflammatory cytokines IL-1 and IL-6 in the serum, ELISA kits were utilized (mlbio, China).

**2.5.5. Echocardiography in pig treated with different particles**

After AMI pigs treated with PMS-siTET3-PEI-PEG/PEI-PEG-CGTArg or PMS-siNC-PEI-PEG/PEI-PEG-CGTArg (N=4), echocardiography was performed one month after the procedure. The pigs were positioned in a supine posture on an examination table, and ultrasound images were captured using an ultrasound device. The echocardiogram encompassed various views, including parasternal long-axis views, parasternal short-axis views, and apical views. These different perspectives allowed for a comprehensive evaluation of cardiac function and morphology. Simpson's method, a well-established technique, was employed to calculate the LVEF from the echocardiographic images.

**2.5.6. TTC and masson staining**

One month after AMI pigs treated with PMS-siTET3-PEI-PEG/PEI-PEG-CGTArg or PMS-siNC-PEI-PEG/PEI-PEG-CGTArg (N=4), the pigs were euthanized and the hearts were collected for further analysis. The hearts were carefully rinsed with a 0.9% NS to remove any external contaminants and then the heart sections were acquired. To assess the infarct size, TTC staining was performed with the aforementioned method.

Furthermore, heart sections were obtained and stained with Masson to assess the extent of cardiac remodeling and fibrosis with aforementioned method.

**2.5.7. Impact on angiogenesis of PMS-siTET3-PEI-PEG/PEI-PEG-CGTArg**

One month after AMI pigs treated with PMS-siTET3-PEI-PEG/PEI-PEG-CGTArg or PMS-siNC-PEI-PEG/PEI-PEG-CGTArg (N=4), the pigs were euthanized and the hearts were collected for immunofluorescence analysis. The staining process was completed with CD31 and VEGFa immunofluorescence staining on heart sections, using the specific antibody anti-CD31 (Proteintech, China) and VEGFa (Proteintech, China).

**2.5.8. Safety Evaluation of PMS-siTET3-PEI-PEG/PEI-PEG-CGTArg in pigs**

Healthy pigs were treated with PMS-siTET3-PEI-PEG/PEI-PEG-CGTArg via the ear vein to further evaluate the in vivo safety of the delivery system. Afterwards, blood samples were collected from the anterior vena cava at various time points: before injection, as well as at 0.5h, 1h, 2h, 24h and 1 week post-injection. The collected samples were used to measure serum parameters, including AST, ALT, Cr, BUN, IL-1 and IL-6;

In addition, pigs were divided into three groups, receiving different treatments: NS treatment for 1 month, PMS-siTET3-PEI-PEG/PEI-PEG-CGTArg treatment for 1 month and repeated injections of PMS-siTET3-PEI-PEG/PEI-PEG-CGTArg for one month (Twice a month); After the treatment, the pigs were euthanized, and the liver, spleen, lungs, kidneys were collected. And the obtained organs were carefully fixed for HE staining.

**2.6 RT-PCR analysis**: Total RNA was extracted with TRIzol reagent (Sigma, USA). cDNA synthesis was carried out with the PrimeScript RT Master Mix Kit (Vazyme, China). RT-PCR was conducted with TB Green Premix Ex Taq Kit (Vazyme, China) and the RT-PCR system (ABI, USA). The relative expression was analyzed using the 2^-ΔΔCt^.

**2.7 WB analysis**: Protein extraction was performed using RIPA lysis buffer and 1% PMSF. The extracted protein was loaded onto a 4-20% gradient gel for electrophoresis. The protein was then transferred onto a PVDF membrane using a transfer system at 350 mA for 60min. Prior to antibody incubation, the membranes were blocked with nonfat milk for 2h. The primary antibody was added and incubated overnight at 4°C, followed by incubation with suitable secondary antibodies conjugated with horseradish peroxidase for 2h at room temperature. After washed for three times with TBST, PVDF membrane was visualized using the enhanced ECL kit and the Molecular Imager ChemiDoc XRS System (Bio RAD). The results were analyzed using Image J software (NIH, Bethesda, USA).

**2.8 Immunofluorescence**: Cells were fixed using paraformaldehyde. Cell permeabilization was achieved with 0.5% Triton X-100 for 10 minutes. After washing with PBS three times for 5 minutes each, cells were blocked with 1% BSA for 30 minutes. Subsequently, the primary antibodies were added, and cells were incubated at room temperature for 1h. After washing with PBS three times for 5 minutes each, the secondary antibody was added and incubation for 30-45 minutes at room temperature. After washing with PBS for 5 minutes, 4',6-diamidino-2-phenylindole (DAPI) staining was performed for 10 minutes. Following three washes with PBS, fluorescent observation was conducted.

**3. Supplementary Statistical Analysis**

Data are presented as mean ± standard deviation (SD) for continuous variables that were normally distributed. For continuous variables that violated the assumptions of normality, data are presented as median with interquartile range (IQR). Categorical variables are summarized as frequency with percentage [n (%)]. For comparisons between groups, independent samples t-tests were used for normally distributed continuous variables with homogeneity of variances. The Mann-Whitney U test (a non-parametric test) was employed when these assumptions were not met. For categorical data, the Pearson chi-square (χ²) test was applied when all expected frequencies were greater than or equal to 5. If any expected frequency was between 1 and 5, either the continuity-corrected chi-square test or Fisher's exact test was used.

In the results, "NS" indicated that there was no statistically significant difference between the groups. "*" denoted a statistically significant difference at a significance level of P < 0.05, "**" represented a highly significant difference at P < 0.01, and "***" indicated an extremely significant difference at P < 0.001. SPSS Statistics 26.0 (IBM, USA) was used for the statistical analysis, and GraphPad Prism 7.0 (GraphPad Software, USA) was utilized for graphical analysis.

[1] Qiu B, Yang E, Zheng Y, et al. Association between SPRY1 and TET3 in skin photoaging and natural aging mechanisms [J]. J Cosmet Dermatol, 2023.

[2] Fu L, Chang H, Wang Z, et al. The effects of TETs on DNA methylation and hydroxymethylation of mouse oocytes after vitrification and warming [J]. Cryobiology, 2019, 90: 41-46.

[3] Zhao M, Hur T Y, No J, et al. Ascorbic acid increases demethylation in somatic cell nuclear transfer embryos of the pig (Sus scrofa) [J]. Asian-Australas J Anim Sci, 2017, 30(7): 944-949.

[4] Kiliszek M, Burzynska B, Michalak M, et al. Altered gene expression pattern in peripheral blood mononuclear cells in patients with acute myocardial infarction [J]. PLoS One, 2012, 7(11): e50054.

[5] Lu W, Xie Z, Tang Y, et al. Photoluminescent Mesoporous Silicon Nanoparticles with siCCR2 Improve the Effects of Mesenchymal Stromal Cell Transplantation after Acute Myocardial Infarction [J]. Theranostics, 2015, 5(10): 1068-1082.

[6] Yan J, Lu X, Zhu X, et al. Effects of miR-26a on Osteogenic Differentiation of Bone Marrow Mesenchymal Stem Cells by a Mesoporous Silica Nanoparticle - PEI - Peptide System [J]. Int J Nanomedicine, 2020, 15: 497-511.

[7] Chen Y, Gu H, Zhang D S, et al. Highly effective inhibition of lung cancer growth and metastasis by systemic delivery of siRNA via multimodal mesoporous silica-based nanocarrier [J]. Biomaterials, 2014, 35(38): 10058-10069.

[8] Zhao J, Ma X L, Ma J X, et al. TET3 Mediates Alterations in the Epigenetic Marker 5hmC and Akt pathway in Steroid-Associated Osteonecrosis [J]. J Bone Miner Res, 2017, 32(2): 319-332.

**Table S1 Primers used for RT-PCR**

| Name | Forward (5’ to 3’) | Reverse (5’ to 3’) |
| --- | --- | --- |
| Homo-TET3 | CTTATGGTCAATGGTGTC | GTTCAGGTTGTTGTTGTA |
| mmu-RTP4 | GAGCCTGCATTTGGATAAGAACA | CCTGCGATTTCAAAGTGTCCG |
| mmu-Snn | CGGGGTGGTAACGGTCATTG | CTCCTTTGTCTCGCCATCAC |
| mmu-Phf11a | CCTGTGCCATGGAAGACCAC | TTCATAGTCACGTTGTGTGGGT |
| mmu-Eva1b | TGCGCACATCAGAGCTAACC | GATGACCAGCAGGCATAGGG |
| mmu- Ifit3 | CCTGTGTACCACAAGGGAACT | CTGGGGCCACACGAAAGAAA |
| mmu- Ier5l | GTGACCACGGTAGAAAACGG | GCCAGGGTAATACTTGCGCTT |
| mmu- Notch1 | GATGGCCTCAATGGGTACAAG | TCGTTGTTGTTGATGTCACAGT |
| mmu- HES1 | GATAGCTCCCGGCATTCCAAG | GCGCGGTATTTCCCCAACA |
| Mmu-DTX2 | CAGCATCCCCTTAGGCCAAG | TGTTCTGGCGAAACTGAGTCC |
