# Supplementary material for: Peptide-functionalized periodic mesoporous silica nanoparticles for monocyte-specific TET3 Silencing enhance cardiac repair after acute myocardial infarction
Source: J Nanobiotechnology. 2025 Nov 26;23:743. doi: 10.1186/s12951-025-03782-4 (PMC12659391; doi:10.1186/s12951-025-03782-4)
Supplement: Supplementary file 1 — Supplementary Material 1. [file 12951_2025_3782_MOESM1_ESM.docx]

**Figure S1**: The peptide validation, the flow cytometry gating strategy, and the transfection efficiency and targeting capability of the nanoparticles. A: Mass spectrometry analysis of the novel peptide; B1-B3: Flow cytometry process for assessing nanoparticle transfection efficiency; B4: Transfection efficiency of particles (10 µg/ml) determined by flow cytometry; B5: Transfection efficiency of particles (20 µg/ml) determined by flow cytometry; B6: Statistical results of transfection efficiency; The flow cytometry gating strategy was designed to select CD14+ (C1) and CD16+ (C2) cells for sorting CD14-CD16+ monocytes and CD14+CD16- monocytes; D1: Gating strategy for leukocyte populations in the blood using flow cytometry antibodies, including CD45⁻ cells, CD45⁺CD11b⁻ lymphocytes, CD45⁺CD11b⁺CD115⁺ monocytes, and CD45⁺CD11b⁺CD115⁻Ly6G⁺ neutrophils. D2: Comparison of nanoparticle uptake by different leukocyte populations in the blood, including CD45⁻ cells, CD45⁺CD11b⁻ lymphocytes, CD45⁺CD11b⁺CD115⁺ monocytes, and CD45⁺CD11b⁺CD115⁻Ly6G⁺ neutrophils

**Figure S2:** The effects of nanoparticles on the distribution of monocytes/macrophages in the infarcted zone and on cardiomyocytes. **A1:** Representative fluorescence images of monocyte/ macrophages distribution in AMI mice treated with PBS, PMS-PEI-PEG/PEI-PEG-CGTArg, PMS-siNC-PEI-PEG/PEI-PEG-CGTArg, PMS-siTET3-PEI-PEG/PEI-PEG-CGTArg; **A2:** Statistical analysis of the fluorescence expression; B: HE staining of heart sections in AMI mice at 30 days after treatments, including PBS, PMS-PEI-PEG/PEI-PEG-CGTArg, PMS-siNC-PEI-PEG/PEI-PEG-CGTArg and PMS-siTET3-PEI-PEG/PEI-PEG-CGTArg;

**Figure S3:** The overexpression analysis of monocytes, the mechanistic investigation of TET3, and the off-target gene analysis of siTET3. A1: Representative immunofluorescence images of TET3 expression in monocytes from AMI (post-treatment) mice after receiving PBS, PMS-PEI-PEG/PEI-PEG-CGTArg, and PMS-PEI-PEG/PEI-PEG-CGTArg (pTET3); A2: Statistical comparison of fluorescence intensity among the different groups; B1: Representative flow cytometry plots of pro-inflammatory monocytes from AMI (post-treatment) mice after receiving PBS, PMS-PEI-PEG/PEI-PEG-CGTArg, and PMS-PEI-PEG/PEI-PEG-CGTArg (pTET3); B2: Statistical comparison of monocyte levels among the different groups. C1: Heatmap analysis of the effect of TET3 expression on the Notch pathway; C2: Assessment of the impact of TET3 expression on Notch pathway–related genes using PCR; D1: Off-target genes of siTET3 presented via heatmap analysis; D2: Off-target genes of siTET3 detected by PCR.

**Figure S4:** Safety of PMS-siTET3-PEI-PEG/PEI-PEG-CGTArg in mice.

Peripheral blood samples were collected at various time points (0, 1, 2, 24, 72 hours and 1week post-injection) for the evaluation of blood biomarkers after mice were treated with PMS-siTET3-PEI-PEG/PEI-PEG-CGTArg, including WBC (A1), RBC (A2), HGB (A3), PLT (A4), ALT (A5), AST (A6), Cr (A7), BUN (A8), IL-1β (A9) and IL-6 (A10); HE staining for major organs in mice treated with different treatments: PBS for one month (B1), PMS-siTET3-PEI-PEG/PEI-PEG-CGTArg for one week (B2), PMS-siTET3-PEI-PEG/PEI-PEG-CGTArg for one month (B3), repeated injections of PMS-siTET3-PEI-PEG/PEI-PEG-CGTArg for one month (B4) (Twice a month), PMS-siTET3-PEI-PEG/PEI-PEG-CGTArg for three month (B5). (N=3, ns p>0.05, *p<0.05, **p<0.01, ***p<0.001)

**Figure S5:** Safety of PMS-siTET3-PEI-PEG/PEI-PEG-CGTArg in pigs.

Safety markers in the blood were evaluated, including ALT (A1), AST (A2), Cr (A3), Bun (A4), IL-1 (A5) and IL-6 (A6); B1: Gross specimens of major organs (including the liver, spleen, lung, and kidney in pigs treated with different treatments: NS for one month (B1), PMS-siTET3-PEI-PEG/PEI-PEG-CGTArg for one month, repeated injections of PMS-siTET3-PEI-PEG/PEI-PEG-CGTArg for one month (Twice a month); Comparisons of major organ weights, including the liver (B2), spleen (B3), lung (B4), and kidney (B5); HE staining for major organs in pigs treated with different treatments: NS for one month (C1), PMS-siTET3-PEI-PEG/PEI-PEG-CGTArg for one month (C2), repeated injections of PMS-siTET3-PEI-PEG/PEI-PEG-CGTArg for one month (C3) (Twice a month). (N=3, ns p>0.05, *p<0.05, **p<0.01, ***p<0.001)
